# Associations of back muscle endurance with occupational back muscle activity and spinal loading among subsistence farmers and office workers in Rwanda

**DOI:** 10.1371/journal.pone.0309658

**Published:** 2024-11-04

**Authors:** Benjamin E. Sibson, Alexandra R. Harris, Andrew K. Yegian, Aimable Uwimana, Assuman Nuhu, Alec Thomas, Dennis E. Anderson, Robert M. Ojiambo, Daniel E. Lieberman

**Affiliations:** 1 Department of Human Evolutionary Biology, Harvard University, Cambridge, MA, United States of America; 2 Division of Basic Sciences, University of Global Health Equity, Butaro, Rwanda; 3 Department of Physiotherapy, University of Rwanda, Kigali City, Rwanda; 4 Institute of Sports Science, University of Lausanne, Lausanne, Switzerland; 5 Center for Advanced Orthopedic Studies, Beth Israel Deaconess Medical Center, Boston, MA, United States of America; 6 Department of Orthopedic Surgery, Harvard Medical School, Boston, MA, United States of America; Ningbo University, CHINA

## Abstract

Over the course of the physical activity transition, machines have largely replaced skeletal muscle as the source of work for locomotion and other forms of occupational physical activity in industrial environments. To better characterize this transition and its effect on back muscles and the spine, we tested to what extent typical occupational activities of rural subsistence farmers demand higher magnitudes and increased variability of back muscle activity and spinal loading compared to occupational activities of urban office workers in Rwanda, and whether these differences were associated with back muscle endurance, the dominant risk factor for back pain. Using electromyography, inertial measurement units, and OpenSim musculoskeletal modeling, we measured back muscle activity and spinal loading continuously while participants performed occupational activities for one hour. We measured back muscle endurance using electromyography median frequency analysis. During occupational work, subsistence farmers activate their back muscles and load their spines at 390% higher magnitudes and with 193% greater variability than office workers. Partial correlations accounting for body mass show magnitude and variability response variables are positively associated with back muscle endurance (*R* = 0.39–0.90 [*P* < 0.001–0.210] and *R* = 0.54–0.72 [*P* = 0.007–0.071], respectively). Body mass is negatively correlated with back muscle endurance (*R* = -0.60, *P* = 0.031), suggesting higher back muscle endurance may be also partly attributable to having lower body mass. Because higher back muscle endurance is a major factor that prevents back pain, these results reinforce evidence that under-activating back muscles and under-loading spines at work increases vulnerability to back pain and may be an evolutionary mismatch. As sedentary occupations become more common, there is a need to study the extent to which occupational and leisure time physical activities that increase back muscle endurance helps prevent back pain.

## Introduction

Levels of physical activity (PA) have been changing worldwide as people undergo the “physical activity transition,” in which people from rural areas move to urban areas where many occupations involve much less PA [1, 2]. In Rwanda for example, rural subsistence farmers do most of their labor by hand, including digging, planting, and harvesting; acquiring and transporting water, fuel and food; and traveling primarily by walking. In contrast, urban Rwandans increasingly have sedentary occupations that involve sitting for most of the day; they acquire food mostly in stores; get water from taps; and use motorized vehicles to travel long distances. In addition, industrial, commercial, and service work common in urban areas is often characterized by constant and repetitive motions with limited variability. According to the World Bank, the number of people in Rwanda living in urban areas increased by 2 million (132%) between 2002 and 2015 which compounds the PA transition.

Differences in PA due to the physical activity transition can be substantial. According to step count and heart rate data, hunter-gatherers and subsistence farmers in small-scale societies typically walk 2–3 times as much per day as average Americans [3] and engage in 10 times as much daily moderate-to-vigorous PA [4–6]. Since PA generates mechanical loads on skeletal and muscle tissue, it is reasonable to infer that declining PA levels associated with urbanization also impact spinal health. In particular, low levels of PA and spinal loading may result in the development of weak back tissues with low endurance that are poor at stabilizing the spine, increasing risk of back pain and injury [7]. This hypothesis is partially supported by evidence that lower back muscle endurance increases an individual’s risk for first-time back pain by three-fold [8, 9], physically active subsistence farmers have higher back muscle endurance compared to more sedentary urbanites [10, 11], and moderate exercise is an effective treatment for back pain [12–14]. On the other hand, conflicting findings that static back endurance is not predictive of back pain [15] and evidence that spinal loading in occupational tasks can be associated with back pain in industrialized populations [16, 17] suggest this hypothesis requires more rigorous testing.

Considerations of connections to back pain and injury have motivated attempts to understand spinal loading and back muscle activity during occupational and leisure time activities. For example, an analysis of loads measured from instrumented vertebral body replacements during activities of daily living found that moving the center of mass of the upper body anteriorly, such as when lifting a weight from the ground, resulted in the highest resultant forces, albeit with large intra- and interindividual variation [18]. However, direct *in vivo* measurements of spinal loads are highly invasive, so it is more feasible to estimate loads using inverse dynamics methods that integrate kinetic data (e.g., ground reaction force) and kinematic data from optical motion capture or inertial measurement unit (IMU) systems [19–24]. It is also increasingly common for researchers to develop and validate musculoskeletal (MSK) models of the thoracolumbar spine that incorporate participant-specific anatomical details and kinematics to estimate non-invasively spinal loading [25–31]. In static postures involving little spinal flexion, activity of major back muscles such as lumbar erector spinae (ES) is linearly related to the load acting on intervertebral joints [32]. However, for postures involving substantial spinal flexion or dynamic movements, the relationship between force and muscle activity is often non-linear and affected by muscle length [33], contraction velocity [34], muscle fatigue [35], and time of day [36]. In postures involving full spinal flexion, surface electromyography (EMG) shows back muscles are not actively contracting yet are experiencing tensile forces (the “flexion-relaxation” phenomenon) [37] and intervertebral joints are subjected to significant loading [38]. Also, co-contraction of trunk muscles increases spine stiffness, hence stability, and may occur independently of any change in load on intervertebral joints [39, 40], further highlighting the importance of considering back muscle activity during occupational activities.

Despite the importance of measuring back muscle activity and spinal loading during occupational activities, the overwhelming majority of such data come from high-income, industrialized regions. A chief reason for this bias is that collecting kinematic and kinetic data requires specialized, often expensive, infrastructure, hardware, and software generally unavailable or impractical in nonindustrialized regions, particularly those with unreliable electricity. Moreover, a key motivation for quantifying back tissue stress has been implementing strategies to better prevent injury among workers and others in high-income, industrialized countries [41–45]. Yet our current understanding of back muscle activity and spinal loading during occupational activities among humans broadly is limited by the paucity of data from rural, nonindustrialized regions that have not yet fully undergone the physical activity transition. Such data has the potential to elucidate better the effects of occupational PA on back function, and in particular back muscle endurance.

The purpose of this study is to test for associations of back muscle endurance with occupational back muscle activity and spinal loading in two regions on opposite sides of a rapid physical activity transition, namely rural subsistence farmers and office working urbanites in Rwanda. Our main aim was to characterize the extent to which occupational activities of subsistence farmers involve higher magnitudes and increased variability of back muscle activity and spinal loading compared to office workers. Our main hypotheses were that i) the magnitude and ii) the variability of spinal loads and back muscle activations during occupational activities is positively associated with back muscle endurance.

## Materials and methods

### Participants

Eight subsistence farmers (4 F, age = 34 ± 7 yrs. [mean ± SD], body mass = 62.1 ± 8.2 kg, height = 1.64 ± 0.04 m; 4 M, age = 40 ± 9 yrs., body mass = 54.9 ± 5.6 kg, height = 1.67 ± 0.09 m) and six office workers (4 F, age = 36 ± 15 yrs., body mass = 67.1 ± 12.1 kg, height = 1.64 ± 0.09 m; 2 M, 28 ± 5 yrs., body mass = 82.7 ± 16.9 kg, height = 1.84 ± 0.02 m) participated in the study. The recruitment period began on June 17, 2023, and ended on July 9, 2023. Subsistence farmers were from the towns of Mugera, Gitanda, Cyasenge, or Rusomo in the rural community of Buyanga, which is located in the Butaro sector in the Burera District of the Northern Province of Rwanda. Informed written consent authorizing the conduction of research in Burera District was provided by the Executive Secretary of Burera District. Farmers were recruited via word of mouth by the Director of Community Development (AU) at the University of Global Health Equity in Butaro. Office workers were from the University of Rwanda in Kigali City, the capital of the Kigali Province of Rwanda. Office workers were recruited via word of mouth by the Deputy Dean of the School of Health Sciences (AN) at the University of Rwanda.

Each participant’s height and body mass were measured; age was self-reported. All participants gave their written informed consent with the help of study team translators who spoke Kinyarwanda, English, and French. Exclusion criteria were being pregnant, having a history of significant MSK injury, any recent serious infectious illness such as malaria, or any chronic illness such as type 2 diabetes or heart disease. Participants who completed any part of the study were compensated with 500 Rwandan francs (about $5). All data collection procedures were approved by the Rwanda National Research Ethics Committee, the Executive Secretary of Burera District, and the Committee on the Use of Human Subjects at Harvard University.

### Back muscle endurance

**Fig 1:**
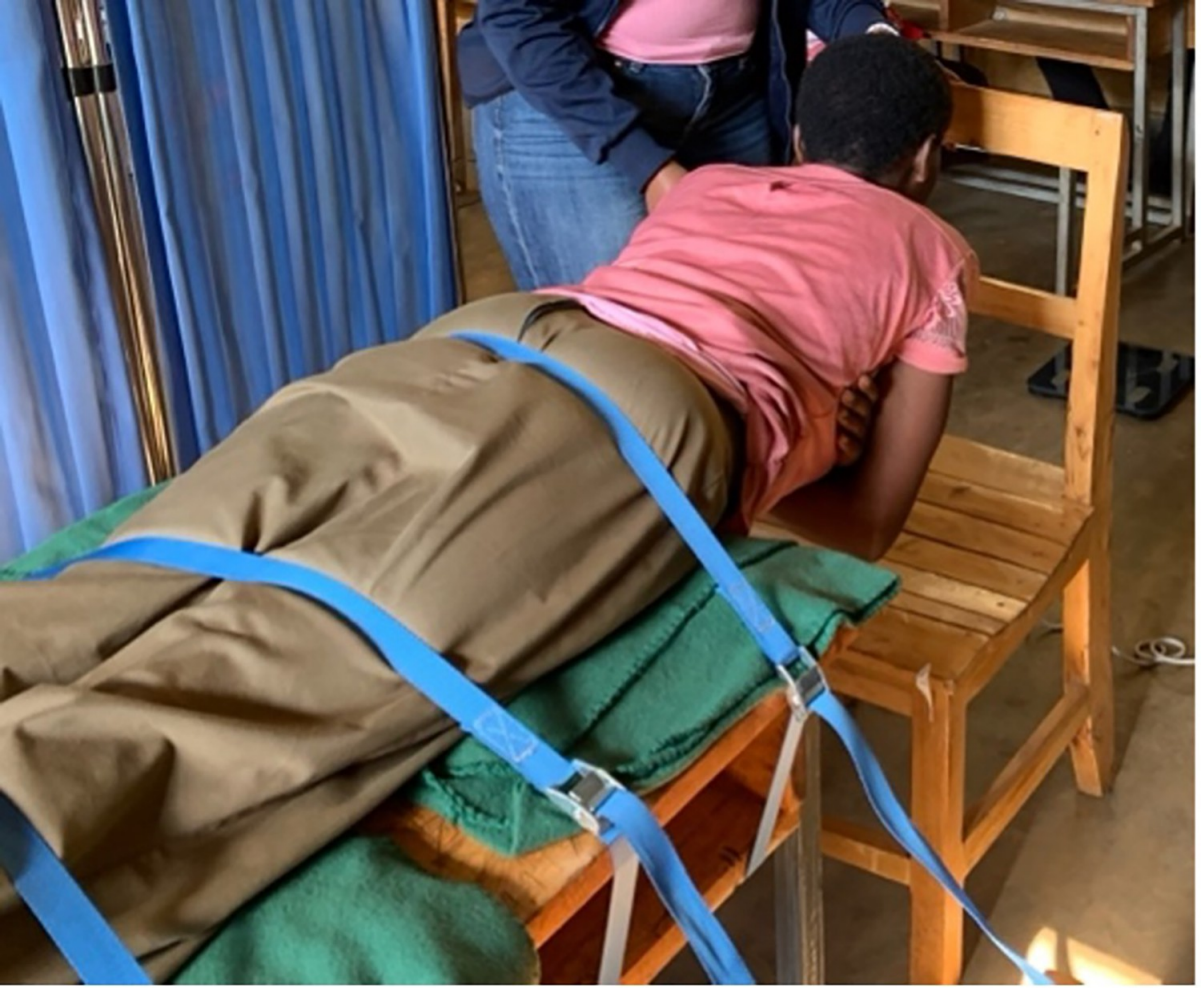
Back endurance test.

Back muscle endurance was measured as the rate of decline of the median frequency (MF) of the EMG power spectrum of the lumbar ES, a primary lower back extensor and stabilizer [46, 47], during a submaximal back endurance test (also known as a Biering-Sorensen test) [8]. The back endurance test is a validated exercise test with high day-to-day reliability that measures endurance of lower back muscles during an isometric contraction [8, 48–52]. Participants were asked to lie prone on a table with their anterior superior iliac spines positioned at the edge of the table and their upper body cantilevered beyond the table’s edge. Before the test, participants were instructed to rest their arms on a chair to support their upper bodies. The lower limbs were firmly secured to the table via three straps wrapped around the buttocks, hamstrings, and calves. Testing commenced once participants were asked to remove their arms from the chair, to cross their arms over their chest (placing each hand on the opposite shoulder), and to maintain their unsupported upper body in a horizontal position for one minute, recorded using a digital stopwatch (Fig 1). Participants were encouraged to hold this position for one minute but were told they could stop the test at any time for any reason. The test was stopped after one minute, when a participant placed their hands on the chair before one minute had elapsed, or if their upper body visibly sank below horizontal for more than three seconds. Verbal feedback was given to help participants maintain their upper body in a horizontal position. Two participants were unable to hold the test position for one minute (1 subsistence farmer, 45 sec; 1 office worker, 26 sec).

Muscle fatigue causes a spectral shift in the EMG power spectrum to lower MF as fast-twitch motor units cease firing and motor unit action potential conduction velocity decreases, resulting in a linear decline in MF over time [53]. A smaller rate of MF decline corresponds to higher muscle endurance. This method of measuring back muscle fatigue has been validated for back endurance tests of one minute, as rate of MF decline during submaximal tests (one minute duration) is significantly positively correlated with endurance times during maximal tests [50, 53–58]. Surface EMG electrodes (Noraxon USA, Scottsdale, AZ, USA) were attached overlying the lumbar ES 2 cm bilateral to the L3 spinous process, with an inter-electrode distance of 20 mm. The skin was cleaned with isopropyl alcohol prior to electrode application. Signals were sampled at 2,000 Hz and bandpass filtered 10–500 Hz (MR3.18, Noraxon USA, Scottsdale, AZ, USA). MF, defined as the frequency that divides the EMG power spectrum into two regions of equal spectral power density [55], was computed at 1 sec sampling periods using a Fast-Fourier Transform algorithm (MATLAB, The MathWorks, Inc., Natick, MA, USA). Following standard convention, the slope of the linear regression of MF over time was normalized to the intercept [54]. After the back endurance test, athletic tape was applied over the electrodes to further secure them in place for continuous recording of back muscle activity during work. Signals were additionally full-wave rectified, averaged between right and left sides, and normalized by dividing by the average value of 10 sec of walking for each participant [59, 60].

### OpenSim musculoskeletal model

An OpenSim 4.4 [61] Thoracolumbar Spine and Ribcage model was used to estimate spinal kinetics. The full-body model includes 598 Hill-type muscle fascicles, 108 degrees of freedom, a fully articulated thoracolumbar spine (vertebrae T1-L5) with 3 rotational and 3 translational degrees of freedom at each joint, and ribcage (24 individual ribs plus a sternum) [62]. The model also includes a combined head and neck body and upper extremities. All major lumbar spine, thoracic spine, and abdominal muscle groups are incorporated. Trunk muscle cross-sectional areas and positions in the model were matched to measurements of 51 men (base male models) and 49 women (base female models) from the Framingham Heart Study [63]. The model has been previously validated for estimates of static and dynamic spine tissue loading and trunk muscle tension against indirect *in vivo* measurements of intradiscal pressure and vertebral compression force from telemeterized implants [22, 28, 61–70]. Participant-specific models were created using the OpenSim Scaling Tool to adjust gender-matched base models to each participant’s measured height and body mass.

Subsistence farmers sometimes carried objects, including bushels of grass or sorghum or 20 L plastic barrels of water in one or both hands or on the head, or carried and used a garden hoe, pickaxe, or shovel with one or both hands. To better account for the inertial effects of these objects on estimates of spinal kinetics, a rigid-body was attached to the hand or head body segments in the OpenSim models. For activities involving one hand or head carrying, the object’s measured mass and rotational inertial properties were assigned to the rigid body. Rotational inertial properties for the bushels and the 20 L plastic barrel were estimated by assuming that each object was cylinder-shaped [71]:

$$I_{XX}=I_{YY}=\frac{1}{12}m(r^{3}+h^{2});\;I_{ZZ}=\frac{1}{2}mr^{2}$$ (1)

where *m* is the mass of the object, *r* is the radius of the object, *h* is the height of the object, and *I*_*XX*_, *I*_*YY*_, and *I*_*ZZ*_ are diagonal elements of the rigid body inertia tensor. Rotational inertial properties for a garden hoe, pickaxe, and shovel held at one end (e.g., during swinging) or in the middle (e.g., during walking) were estimated by assuming that the object had the geometry of a slender rod [71]:

$$I_{XX}=I_{ZZ}=\frac{1}{3}mh^{2};\;I_{YY}=0$$ (2)

$$I_{XX}=I_{ZZ}=\frac{1}{12}mh^{2};\;I_{YY}=0$$ (3)

For activities involving holding an object in both hands, half the mass and estimated rotational inertial properties of the object were assigned to each rigid body. This modeling approach was chosen for ease of implementation [72] and has been shown to result in satisfactory residual forces [22, 73]. Properties of each object are presented in Table 1.

**Table 1:** Physical properties of objects used as rigid-bodies in OpenSim models.

Object	Geometry	Mass (kg)	Radius (m)	Height (m)	I_XX_	I_YY_	I_ZZ_
20 L plastic barrel of water	Cylinder	20.0	0.14	0.37	0.326	0.196	0.326
Grass bushel	Cylinder	6.4	0.24	0.20	0.110	0.177	0.110
Sorghum bushel	Cylinder	25.4	0.24	0.20	0.435	0.701	0.435
Garden hoe/pickaxe/shovel (held at end)	Slender rod	1.6	n/a	1.53	0.619	0	0.619
Garden hoe/pickaxe/shovel (held in middle)	Slender rod	1.6	n/a	1.53	0.310	0	0.310

### Spinal kinematics

To measure spinal kinematics, IMU sensors (Noraxon USA, Scottsdale, AZ, USA) were affixed to participants with either Velcro straps or double-sided adhesive tape at eight upper body locations: over the T2 spinous process, the L1 spinous process, the pelvis (the body area of the sacrum), midway between both shoulder and elbow joints (on the lateral humeri), both posterior and distal forearms (where there is a low amount of muscle tissue), and the back of the head. IMU trajectories were sampled at 200 Hz (MR3.18, Noraxon USA, Scottsdale, AZ, USA). An initial IMU calibration required participants to stand still, facing forward with arms at the sides, for 10 sec. IMU sensors were recalibrated every 10 min during data recording to mitigate signal drift.

We used the OpenSense workflow in OpenSim [74–76] to convert IMU kinematics to the OpenSim world frame. First, the OpenSim IMU Placer Tool assigned each IMU sensor to the appropriate body of each participant-specific model during the calibration pose of the model. Next, using models with kinematic constraints limiting the spine to three independent degrees of freedom [69], the OpenSim IMU Inverse Kinematics Tool tracked quaternion orientation data from each IMU sensor, finding the pose of the model at each time-step that minimized the difference between the orientation data from the IMU sensors and IMU frames on the calibrated OpenSim model. Because Noraxon MR3.18 software does not provide pelvis position data in the absence of foot IMU sensors, we estimated pelvis position in the OpenSim world frame by filtering the pelvis IMU accelerations with a low-pass, zero-phase, second-order 30 Hz Butterworth filter, integrated the signal twice, and then applied a high-pass, zero-phase 1 Hz Butterworth filter to remove drift error that resulted from the double integration [23]. IMU inverse kinematics coordinates were filtered with a low-pass, zero-phase, fourth order 4 Hz Butterworth filter [53] prior to performing kinetic analyses.

### Continuous data recording during occupational activities

After completing the back endurance test and donning the IMU sensors, participants were asked to perform the occupational activities they most commonly engage in, for one hour. At least 15 min of time elapsed between the conclusion of the back endurance test and the start of data recording, which is enough time for the lower back extensors to fully recover from fatigue [77]. Because successful data recording required the sensors be kept within 20 m of the sensor receiving device, to troubleshoot potential issues with data recording in real time, and to document activities performed by participants, the first author and one study team translator accompanied each participant for the full duration of sensor wear-time in a focal follow. Focal sampling data were collected for one participant at a time [78] and were predetermined to be approximately 60 min to provide adequate time for several common occupational activities to be performed continuously. All focal samples began in the morning between 9:00–10:00 AM. During and immediately after each focal sample, the initial activity state (e.g., standing, walking), the transition times between activity states, and the activity states following each subsequent transition were recorded, as were any objects handled [78]. Across participants, EMG sensor data recordings were 52.5 ± 13.9 min in duration and IMU sensor data recordings were 46.4 ± 16.0 min in duration.

### Spinal kinetics

The OpenSim model used a top-down modeling approach to estimate spinal kinetics, with segment analysis commencing at the distal segments (e.g. hands) and working through the kinetic chain to the lumbosacral joint (L5/S1). The OpenSim Inverse Dynamics Tool calculated net joint moments in flexion-extension (FE), lateral bending (LB), and axial rotation (AR) at each thoracolumbar joint (T1/T2 –L5/S1, *N* = 17 joints) from the IMU inverse kinematics coordinates. Moments were made dimensionless by dividing by the product of body weight and height. The OpenSim Static Optimization Tool estimated the model muscle forces that can produce the calculated net joint moments by minimizing the total cubed activations of all musculotendon actuators. The OpenSim Joint Reaction Analysis Tool was used to calculate the combined effect of reactionary and muscle forces on thoracolumbar net joint compression, anteroposterior (AP) shear, and mediolateral (ML) shear forces. Net reaction forces were made dimensionless by dividing by body weight. Absolute values were used for analyses to correct for the values switching signs from positive to negative. Vector magnitudes (VM) were computed as the square root of the sum of squares in each anatomical plane:

$$VM=\sqrt{{\left(FE\;or\;compression\right)}^{2}+{\left(LB\;or\;AP\;shear\right)}^{2}+{\left(AR\;or\;ML\;shear\right)}^{2}}$$ (4)

### Statistical analysis

All statistical procedures were performed using The Statistics and Machine Learning Toolbox (MATLAB, The MathWorks, Inc., Natick, MA, USA). Significance level was set to *α* = 0.05 for all tests. Given the small sample size, no outliers were removed from analyses. In order not to assume normality, two-tailed nonparametric Wilcoxon rank-sum tests (also known as Mann-Whitney *U* tests) were used to test for differences in age, body mass, and height between groups (subsistence farmer [Rural] or office worker [Urban]). To characterize the extent to which occupational activities of subsistence farmers involved higher magnitudes and variability of back muscle activity and spinal loading, we used general linear models (GLM) with different response variables, which we describe next.

Considering magnitudes of back muscle activity and spinal loading during occupational work, GLMs were used to compare between groups the root mean square (RMS) of lumbar ES activity, net joint moment, and net joint reaction force time-series data. In addition, GLMs were used to compare between groups the proportion of data recording spent with lumbar ES activity, net joint moment, and net joint reaction force magnitudes higher than twice the average value measured during walking. We chose walking as our threshold PA because it is the most fundamental form of light-moderate PA among humans [3]. To calculate this proportion, probability density functions were fit to the time-series data using the *histcounts* function in MATLAB. We defined the proportion of data recording, or time, spent with magnitude response variable values higher than twice the average value during walking as the area under the probability density function above twice the average walking value, divided by the total area. Considering variability of back muscle activity and spinal loading during occupational work, GLMs were used to compare between groups the interquartile range (IQR) and standard deviation (SD) of lumbar ES activity, net joint moment, and net joint reaction force time-series data. All variables were log-transformed to achieve normality. The key predictor variable was group (Rural or Urban), with age, body mass, and sex included as covariates in the GLMs:

$$log(variable)∼\beta_{0}+\beta_{1}(age)+\beta_{2}(body\;mass)+\beta_{3}(sex)+\beta_{4}(group)+\epsilon$$ (5)

For each response variable, we present sample group means, standard deviations, and percentage differences between groups. Type-3 ANOVAs were performed on model variance to test for significance of model effects. For all models, residual plots were used to check for homoscedasticity. To assess statistical power, we conducted a *post-hoc* power analysis using G*Power Version 3.1.9.6 using the RMS of net lumbosacral joint reaction force time-series data.

To test the hypothesis that the extent to which individuals load their spines and activate their back muscles during work is associated with back muscle endurance, Pearson’s product-moment partial correlations (*R*) and coefficients of determination (*R*^*2*^) were used to test for associations between each log-transformed response variable with normalized slopes of MF decline, accounting for the effect of body mass. Partial correlations with body mass were used because there is some evidence that body mass can be independently associated with back muscle endurance [79]. For brevity, we consider only L5/S1 VM for moment and reaction force correlational analysis. Effect sizes for *R* were based on Cohen’s criteria (0.7 = large, 0.5 = moderate, 0.3 = small) [80]. Issues with the EMG equipment rendered us unable to collect MF data during the back endurance test for one participant.

## Results

Full sample characteristics for anthropometrics, presented in Table 2, show that subsistence farmers and office workers were of similar age, height, and body mass.

**Table 2:** Anthropometric data.

	Rural (*N* = 8)		Urban (*N* = 6)			
Variable	Mean	SD	Mean	SD	%	*P*
**Age (years)**	37	8	33	13	11	0.394
**Body mass (kg)**	60.7	6.7	72.3	14.4	-16	0.088
**Height (cm)**	165.5	6.5	170.8	12.5	-3	0.509

% = percentage difference between groups. *P*-values are for Wilcoxon rank-sum tests.

### Occupational back muscle activity magnitude and variability

**Fig 2:**
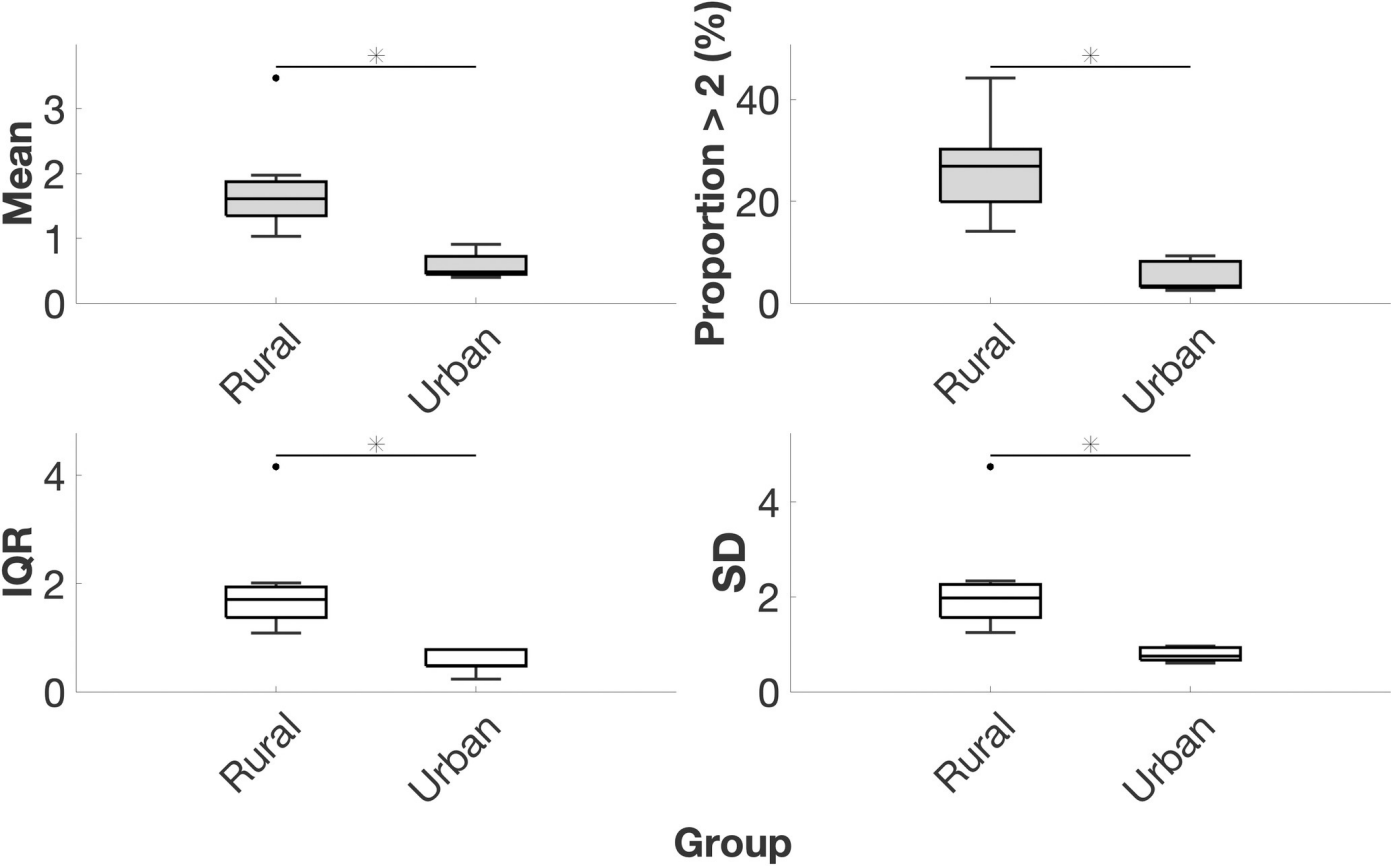
Boxplots of lumbar erector spinae activity mean, proportion over twice walking value, interquartile range, and standard deviation. Data are normalized to the average value measured during 10 sec of walking. >2 = greater than twice walking value; IQR = interquartile range; SD = standard deviation; Middle horizontal bar is the median, upper and lower horizontal bars are the 75^th^ and 25^th^ percentiles, upper and lower vertical bars are maximum and minimum values, and dots are potential outliers. * denotes *P* < 0.05 for group variable in GLM.

The occupational activities of subsistence farmers involved 209% higher mean lumbar ES activity (*P* = 0.003) and 430% higher proportion of time spent with lumbar ES activity greater than twice walking value (*P* < 0.001) compared to office workers. Occupational activities of subsistence farmers involved 253% higher IQR of lumbar ES activity (*P* = 0.006) and 182% higher SD of lumbar ES activity (*P* = 0.002; Table 3 and Fig 2 and Fig A in S2 File). Age, body mass, and sex were not significant predictors. Model coefficient estimates and summary statistics can be found in Tables A and B in S2 File.

**Table 3:** Lumbar erector spinae activity mean, proportion over twice walking value, interquartile range, and standard deviation.

	Rural (*N* = 8)		Urban (*N* = 6)			
Variable	Mean	SD	Mean	SD	%	*P*
**Mean**	1.77	0.76	0.57	0.20	209	**0.003**
**Proportion >2 (%)**	26.53	9.39	5.01	2.94	430	**<0.001**
**IQR**	1.91	0.96	0.54	0.21	253	**0.006**
**SD**	2.20	1.09	0.78	0.15	182	**0.002**

Data are normalized to the average value measured during 10 sec of walking. >2 = greater than twice walking value; IQR = interquartile range; SD = standard deviation; % = percent difference between group means. *P*-values are for group variable in GLM.

### Occupational net lumbosacral joint moment magnitude and variability

**Fig 3:**
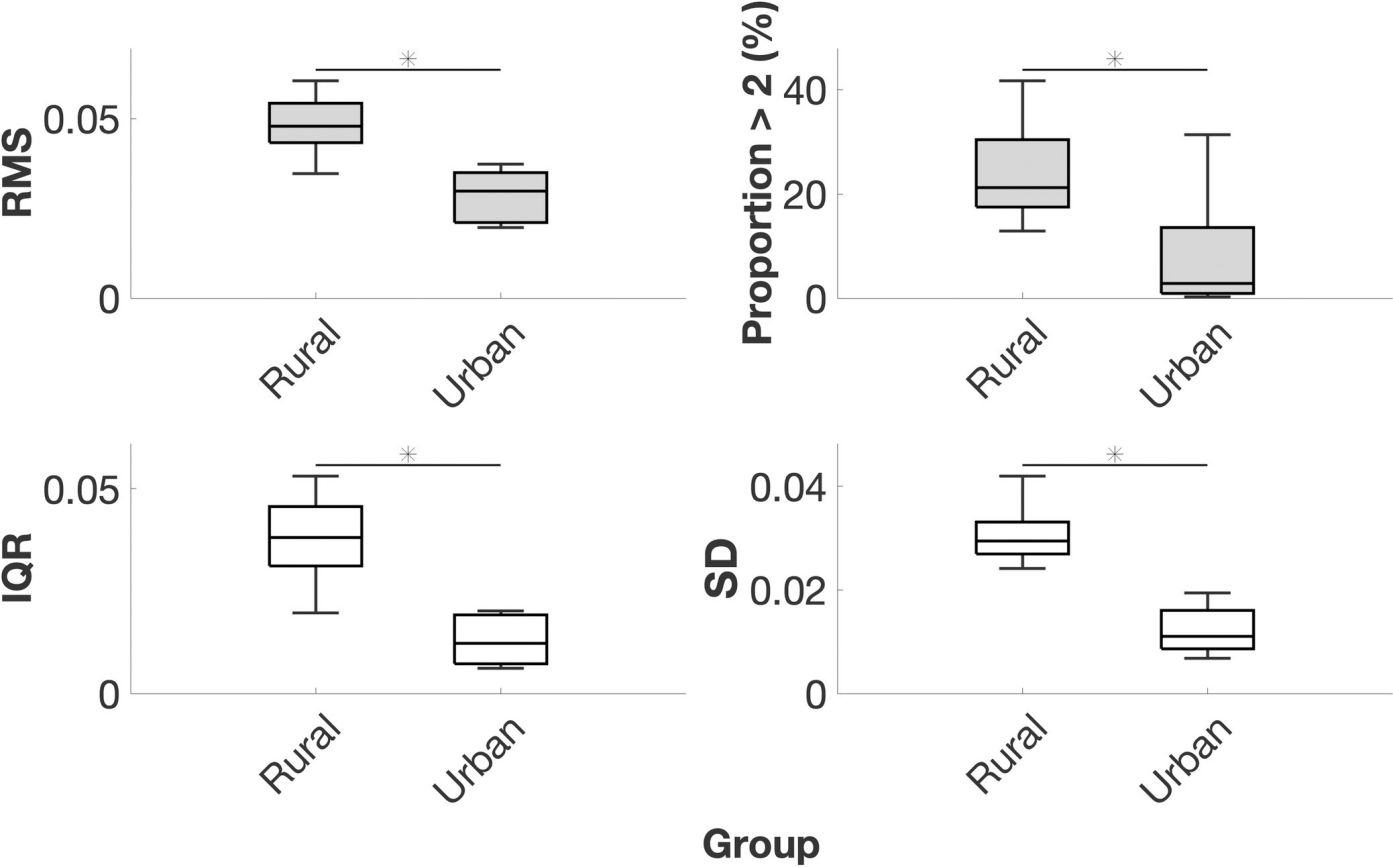
Boxplots of net lumbosacral joint moment vector magnitude root mean square, proportion over twice walking value, interquartile range, and standard deviation. Data are normalized to body weight times height. RMS = root mean square; >2 = greater than twice walking value; IQR = interquartile range; SD = standard deviation. Middle horizontal bar is the median, upper and lower horizontal bars are the 75^th^ and 25^th^ percentiles, upper and lower vertical bars are maximum and minimum values, and dots are potential outliers. * denotes *P* < 0.05 for group variable in GLM.

Considering vector magnitudes (VM), the occupational activities of subsistence farmers involved 68% higher net L5/S1 moment RMS (*P* = 0.018) and 180% higher proportion of time spent with net L5/S1 moment greater than twice walking value (*P* = 0.033). The occupational activities of subsistence farmers were more variable, with 253% higher net L5/S1 moment IQR (*P* = 0.006) and 182% higher net L5/S1 moment SD (*P* = 0.002; Table 4, Fig 3 and Fig B in S2 File). Descriptive statistics and *P*-values for net L5/S1 moments in FE, LB, and AR can also be found in Table 4. Similar results were observed for joints T1 –L5 (descriptive statistics presented in Table C in S2 File). Model coefficients and summary statistics for net L5/S1 moments can be found in Tables D and E in S2 File. Model coefficients and summary statistics for joints T1 –L5 can be found in S3 File. Lower body mass was associated with subjecting L5/S1 to net LB moments greater than twice walking value (*P* = 0.004). Age, body mass, and sex were otherwise not significant predictors.

**Table 4:** Net lumbosacral joint moment root mean square, proportion over twice walking value, interquartile range, and standard deviation.

		Rural (*N* = 8)		Urban (*N* = 6)			
**Variable**	**Axis**	**Mean**	**SD**	**Mean**	**SD**	**%**	***P***
**RMS**	**VM**	0.048	0.009	0.029	0.007	68	**0.018**
	**FE**	0.042	0.009	0.027	0.008	56	0.068
	**LB**	0.019	0.003	0.008	0.002	146	**<0.001**
	**AR**	0.012	0.003	0.004	0.001	189	**<0.001**
**Proportion >2 (%)**	**VM**	24.107	9.484	8.617	12.171	180	**0.033**
	**FE**	27.871	10.252	11.670	15.579	139	**0.017**
	**LB**	8.291	5.773	1.179	0.983	603	**0.008**
	**AR**	9.588	5.477	0.811	0.543	1083	**0.004**
**IQR**	**VM**	0.038	0.011	0.013	0.006	193	**0.005**
	**FE**	0.036	0.012	0.015	0.007	141	**0.025**
	**LB**	0.019	0.004	0.006	0.002	237	**<0.001**
	**AR**	0.010	0.002	0.003	0.001	263	**<0.001**
**SD**	**VM**	0.031	0.006	0.012	0.005	152	**0.001**
	**FE**	0.031	0.006	0.014	0.005	122	**0.005**
	**LB**	0.019	0.002	0.007	0.001	161	**<0.001**
	**AR**	0.012	0.003	0.004	0.001	202	**<0.001**

Data are normalized to body weight times height. RMS = root mean square; VM = vector magnitude; >2 = greater than twice walking value; IQR = interquartile range; SD = standard deviation; FE = flexion-extension; LB = lateral bending; AR = axial rotation; % = percent difference between group means. *P*-values are for group variable in GLM.

### Occupational net lumbosacral joint reaction force magnitude and variability

**Fig 4:**
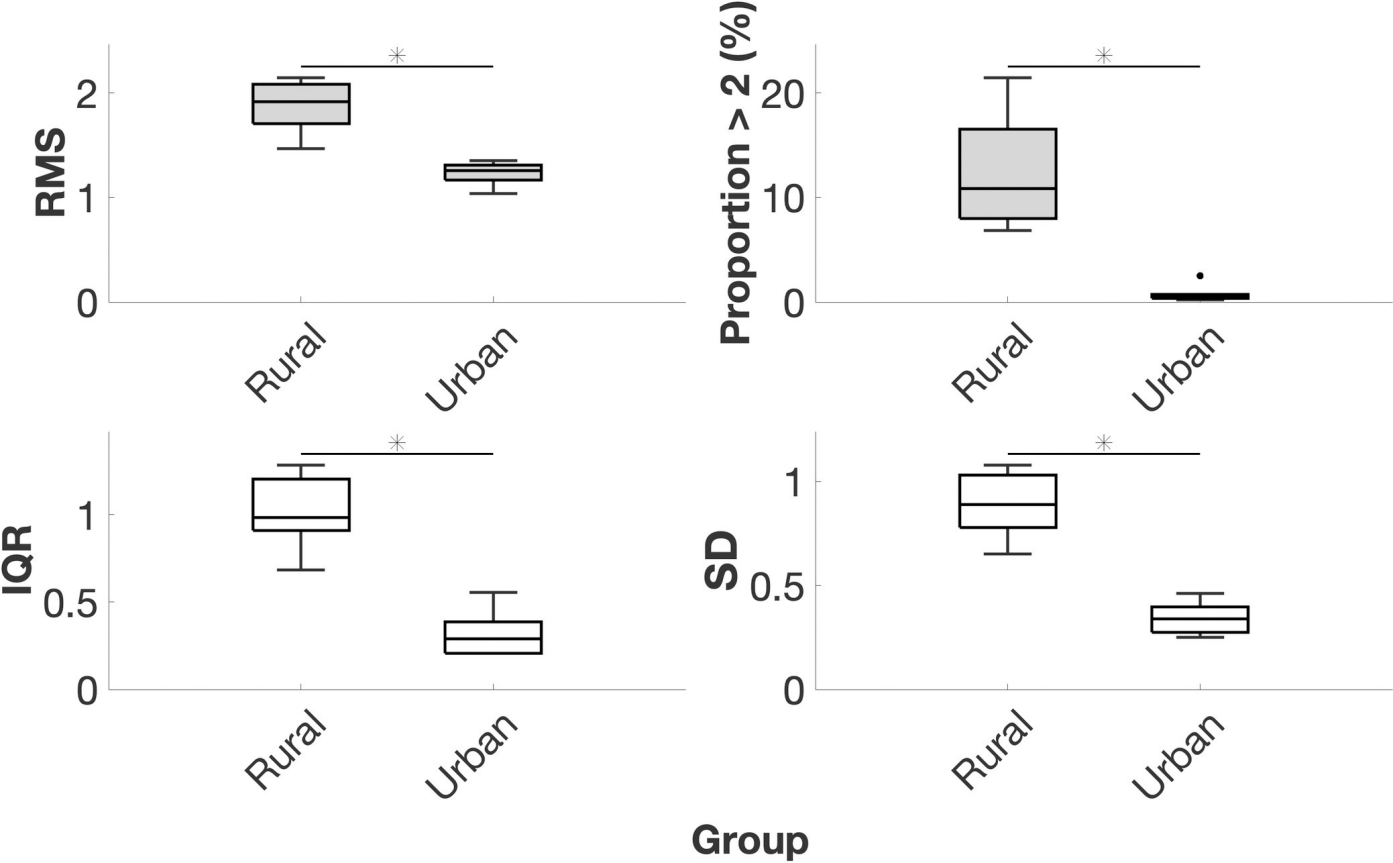
Boxplots of net lumbosacral joint reaction force vector magnitude root mean square, proportion over twice walking value, interquartile range, and standard deviation. Data are normalized to body weight. RMS = root mean square; >2 = greater than twice walking value; IQR = interquartile range; SD = standard deviation. Middle horizontal bar is the median, upper and lower horizontal bars are the 75^th^ and 25^th^ percentiles, upper and lower vertical bars are maximum and minimum values, and dots are potential outliers. * denotes *P* < 0.05 for group variable in GLM.

Considering VM, the occupational activities of subsistence farmers involved 53% higher net L5/S1 reaction force RMS (*P* = 0.001) and 1,401% higher proportion of time spent with net L5/S1 reaction force greater than twice walking value (*P* < 0.001). Again, occupational activities of subsistence farmers involved 216% higher net L5/S1 reaction force IQR (*P* < 0.001) and 159% higher net L5/S1 reaction force SD (*P* < 0.001; Table 5, Fig 4 and Fig C in S2 File). Descriptive statistics and *P*-values for net L5/S1 reaction force in compression, AP shear, and ML shear can also be found in Table 5. We observed similar results for joints T1 –L5 (descriptive statistics are presented in Table F in S2 File). Model coefficients and summary statistics for net L5/S1 reaction forces can be found in Tables G and H in S2 File. Model coefficients and summary statistics for joints T1 –L5 can be found in S3 File. Being of male sex was predictive of higher net L5/S1 AP shear force SD (*P* = 0.018). Age, body mass, and sex were otherwise not significant predictors. Based on the model summary statistics for net L5/S1 reaction force RMS (Table H in S2 File) and our sample size *N* = 14, the statistical power of the test is 0.96, which suggests the probability of making a Type II error is very low.

**Table 5:** Net lumbosacral joint reaction force root mean square, proportion over twice walking value, interquartile range, and standard deviation.

		Rural (*N* = 8)		Urban (*N* = 6)			
Variable	Axis	Mean	SD	Mean	SD	%	*P*
**RMS**	**VM**	1.878	0.241	1.228	0.113	53	**0.001**
	**Comp**	1.813	0.228	1.192	0.111	52	**0.001**
	**AP shear**	0.481	0.088	0.293	0.034	64	**0.001**
	**ML shear**	0.071	0.015	0.035	0.008	103	**0.002**
**Proportion >2 (%)**	**VM**	12.400	5.408	0.826	0.852	1401	**<0.001**
	**Comp**	12.186	4.697	0.787	0.807	1448	**<0.001**
	**AP shear**	14.323	6.013	1.156	1.342	1139	**<0.001**
	**ML shear**	6.169	4.224	1.389	1.199	344	0.074
**IQR**	**VM**	1.020	0.203	0.323	0.133	216	**<0.001**
	**Comp**	0.984	0.192	0.312	0.130	216	**<0.001**
	**AP shear**	0.266	0.070	0.083	0.031	220	**<0.001**
	**ML shear**	0.059	0.015	0.030	0.005	94	**<0.001**
**SD**	**VM**	0.891	0.155	0.344	0.079	159	**<0.001**
	**Comp**	0.857	0.145	0.331	0.078	159	**<0.001**
	**AP shear**	0.240	0.059	0.095	0.019	154	**<0.001**
	**ML shear**	0.069	0.016	0.032	0.009	114	**0.002**

Data are normalized to body weight. RMS = root mean square; VM = vector magnitude; >2 = greater than twice walking value; IQR = interquartile range; SD = standard deviation; Comp = compression; AP = anteroposterior; ML = mediolateral; % = percent difference between group means. *P*-values are for group variable in GLM.

### Associations between back muscle activity, spinal loading, and back muscle endurance

**Fig 5:**
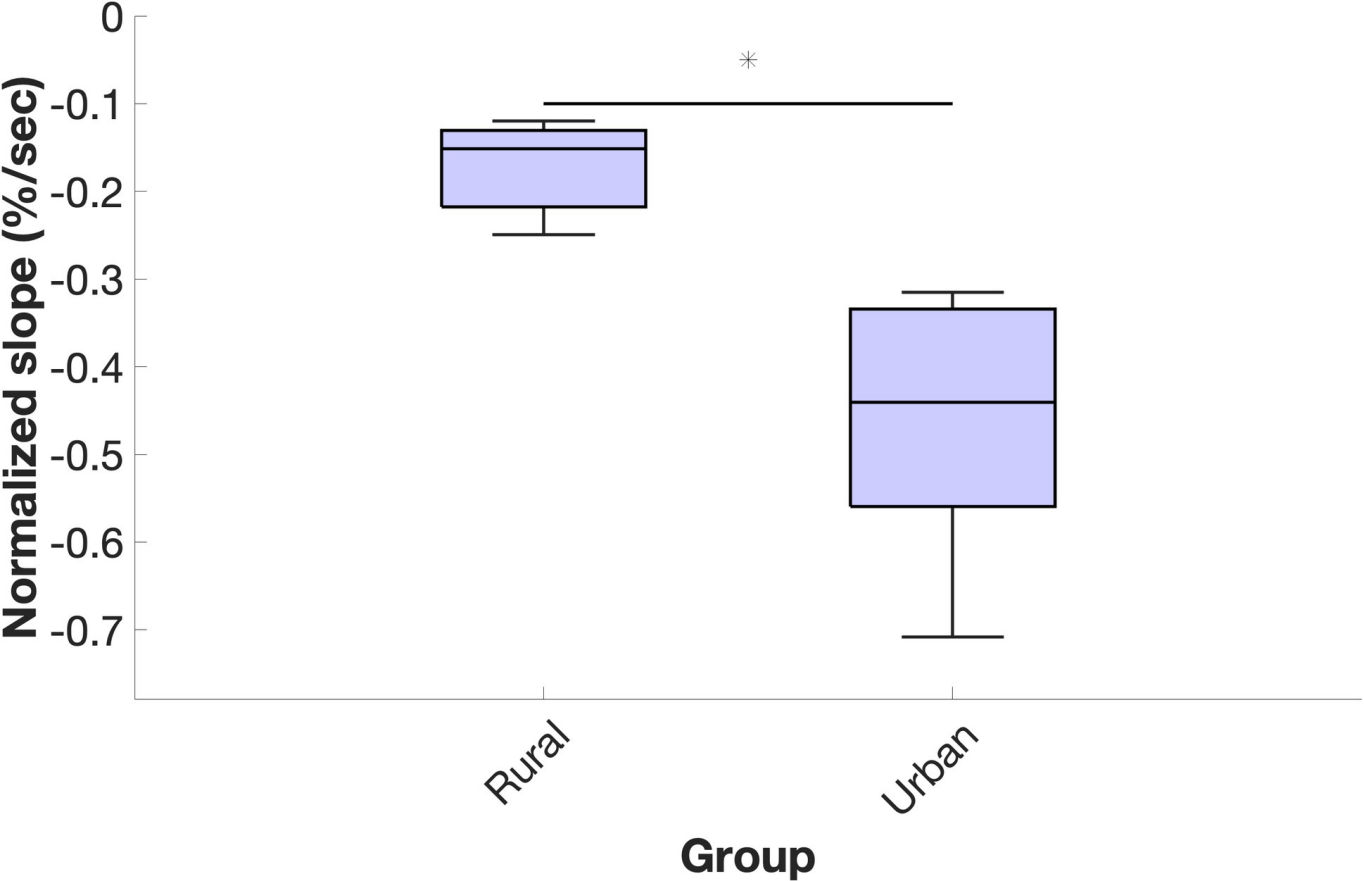
Normalized slope of electromyography median frequency during back endurance test. Middle horizontal bar is median, upper and lower horizontal bars are the 75^th^ and 25^th^ percentiles, and upper and lower vertical bars are maximum and minimum values. * denotes *P* < 0.05 for Wilcoxon rank-sum test.

Subsistence farmers had significantly higher back muscle endurance. There was no difference in MF linear regression intercept between groups (*P* = 0.234), but subsistence farmers had 66% lower MF linear regression slopes compared to office workers (*P* = 0.008), corresponding to 63% lower normalized slopes of MF decline (*P* = 0.001) (Table 6 and Fig 5).

**Table 6:** Electromyography median frequency data.

	Rural (*N* = 7)		Urban (*N* = 6)			
Variable	Mean	SD	Mean	SD	%	*P*
**MF Intercept (Hz)**	81.0	24.0	92.8	22.0	-13	0.234
**MF Slope (Hz/s)**	-0.155	0.092	-0.449	0.220	-66	**0.008**
**Normalized slope (%/s)**	-0.175	0.051	-0.466	0.148	-63	**0.001**

MF = median frequency; % = percentage difference between groups. *P*-values are for Wilcoxon rank-sum tests.

**Fig 6:**
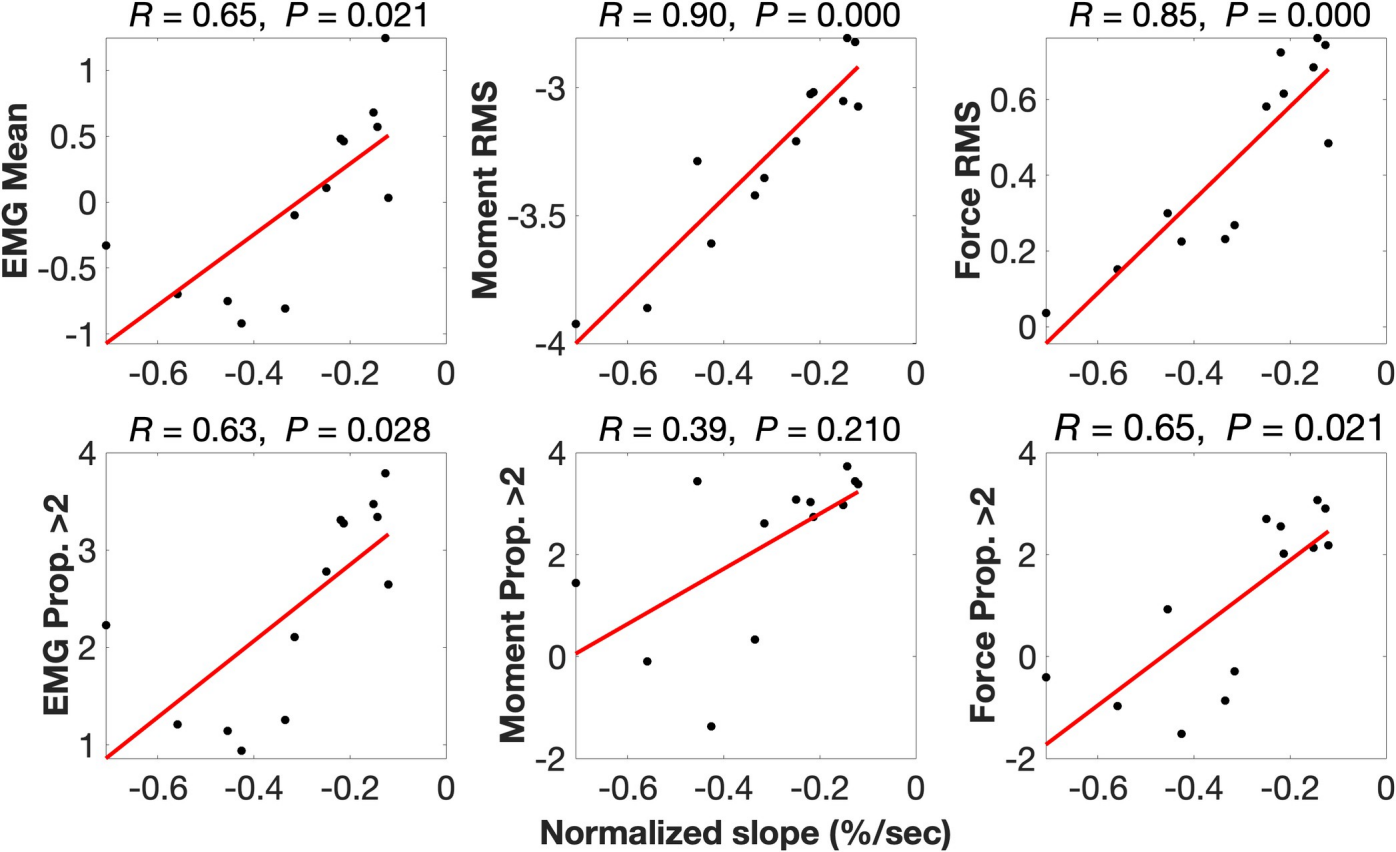
Partial correlations between normalized slope of median frequency decline with magnitude response variables. Partial correlations account for body mass. EMG = lumbar erector spinae activity; RMS = root mean square; Prop. >2 = proportion greater than twice walking value; *R* = Pearson’s product-moment partial correlation coefficient; *P* = significance of the correlation.

**Fig 7:**
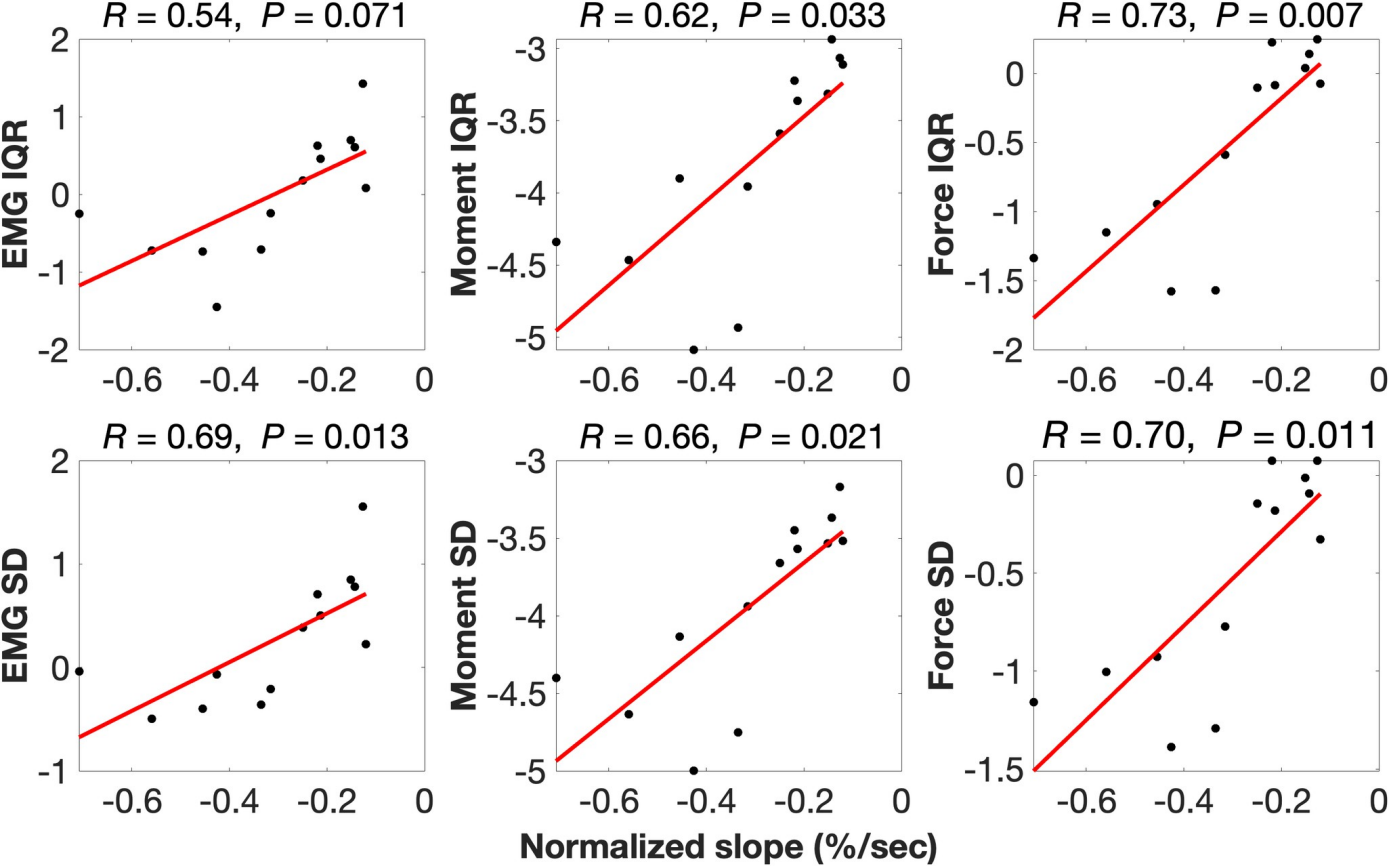
Partial correlations between normalized slope of median frequency decline with variability response variables. Partial correlations account for body mass. EMG = lumbar erector spinae activity; IQR = interquartile range; SD = standard deviation; *R* = Pearson’s product-moment partial correlation coefficient; *P* = significance of the correlation.

Pearson’s product-moment partial correlations (*R*), accounting for body mass, found positive associations ranging from *R =* 0.39–0.90 between normalized slope of MF decline with magnitude-related back muscle activity and spinal loading variables (Table 7 and Fig 6). Similarly, Pearson’s product-moment partial correlations found positive associations ranging from *R =* 0.54–0.73 between normalized slope of MF decline with variability-related back muscle activity and spinal loading variables (Table 7 and Fig 7). Pearson’s product-moment correlation found body mass is negatively associated with normalized slope of MF decline (*R* = -0.60, *P* = 0.031).

**Table 7:** Partial correlations between normalized slope of median frequency decline with response variables.

Data Type	Variable	*R*	*R* ^*2*^	*P*
**Lumbar erector**	**Mean**	0.65	0.42	**0.021**
**spinae activity**	**Proportion > 2 (%)**	0.63	0.40	**0.028**
	**IQR**	0.54	0.29	0.071
	**SD**	0.69	0.48	**0.013**
**Net lumbosacral**	**RMS**	0.90	0.81	**<0.001**
**joint moment vector**	**Proportion > 2 (%)**	0.39	0.15	0.210
**magnitude**	**IQR**	0.62	0.38	**0.033**
	**SD**	0.66	0.44	**0.021**
**Net lumbosacral**	**RMS**	0.85	0.72	**<0.001**
**joint reaction force**	**Proportion > 2 (%)**	0.65	0.42	**0.021**
**vector magnitude**	**IQR**	0.73	0.53	**0.007**
	**SD**	0.70	0.49	**0.011**

Partial correlations account for body mass. RMS = root mean square; >2 = greater than twice walking value; IQR = interquartile range; SD = standard deviation; *R* = Pearson’s product-moment partial correlation coefficient; *R*^*2*^ = coefficient of variation; *P* = significance of the correlation. *N* = 13.

## Discussion

This study characterizes back muscle activity and spinal loading among Rwandan subsistence farmers and office workers during occupational activities and associates these data with a measure of back muscle endurance. As expected, subsistence farmers activate their back muscles and load their spines more intensely and more variably than office workers during typical occupational activities. As hypothesized, ten of the twelve (83%) magnitude and variability response variables are positively associated with back muscle endurance, accounting for the effects of body mass. While it is unsurprising that reduced occupational PA associated with urbanization and increased reliance on machines for mechanical work involves less intense and less variable back muscle activity and spinal loading, these data support the hypothesis that these activities not only reduce loading of the spine but are also associated with reduced back muscle endurance [7]. In addition, back muscle endurance is negatively associated with body mass, suggesting higher back muscle endurance may be also partly attributable to having lower body mass. Because greater back muscle endurance has been shown to help prevent back pain [8, 9], it is likely that the ongoing physical activity transition will lead to increased rates of back pain and injuries as more individuals use their backs less. Since this study used a cross-sectional sample to test for associations, future prospective studies are necessary to test rigorously whether occupational differences in back muscle activity and spinal loading of the sort documented here underlie changes in the prevalence of back pain in societies undergoing urbanization.

Quantifying the prevalence of back pain is difficult due to its multifactorial etiology, varied presentation, and common diagnosis as “nonspecific” [81, 82], but injury resulting in back pain often results from mechanical loading of tissues that exceeds their capacity. The risk and extent of tissue damage depends on the magnitude, rate, direction, and frequency of loading, as well as tissue properties (e.g., fatigue tolerance) [83]. Risk of tissue damage is thus context-dependent, and capacity can be exceeded via loads of absolutely high magnitudes, loads of relatively high magnitudes applied to weak or fatigued tissues, loads of any magnitude applied with high frequency (e.g., overuse) [84], or loads applied constantly and repetitively without much variation [85]. Nevertheless, moderate mechanical loading of the spine is required for proper development and maintenance of tissue structure and function, helping develop capacity to match the demands of PA [86]. This balance has been modeled as a U-shaped curve with minimum risk of injury associated with moderate spinal loading and is supported by evidence that moderate PA is associated with strong, dense vertebrae and fatigue-resistant back muscles capable of stabilizing the spine during various loading scenarios [87–93]. To test this U-shaped model, future work should measure back pain prevalence and occupational and leisure-time spinal loading in groups that vary in subsistence strategy and PA level.

Our results add to other studies of back muscle endurance measured using MF analysis of EMG data. Although most MF studies of the back muscles are of individuals from postindustrial countries [49, 54, 94, 95], the Rwandan data here complement a 2021 study that used MF analysis of EMG data to compare back muscle endurance between subsistence farmers and urbanites from the same Kalenjin ethnolinguistic group in Kenya [10]. There, a group difference was observed for women but not men, which the authors speculated was because in Kalenjin culture only women perform head carrying of water and firewood. Our observation is that women in the rural region of Rwanda where we conducted this study generally also perform more head carrying of water than men. Although our sample size (*N* = 14) was smaller than the 2021 study in Kenya (*N* = 74) and consisted of only two male office workers, the fact that we did not observe a similar sex difference may suggest that other occupational or leisure time activities performed by men compensated enough to still give them high back muscle endurance. The subsistence farmers in this study had higher EMG-determined back muscle endurance compared to most values in the literature with the exception of the female Kenyan subsistence farmers described above, while office workers had comparable values [10, 54, 94, 95]. Given the reduced back muscle endurance we observed for office working urbanites, it appears their leisure time PA habits did not compensate for the lack of intense and variable back muscle activity and spinal loading patterns they experienced during work.

Our results also add to previous studies on occupational back muscle activity and spinal loading. In general, the lumbar ES activity (Table 3 and Fig 3) and spinal loading magnitudes (Tables 4 and 5; Figs 3 and 4) we report fall within the range of published values for workers in postindustrial environments such as firefighters [41], U.S. Marines [42], competitive weightlifters [43], manual materials handlers [44, 45], computer typists [96], and airport baggage handlers [97]. The values we report also fall within the range of published values of spinal loading during activities of daily living like opening windows [18, 65] and more generalized activities like sitting [98, 99], walking [19, 20, 23, 100, 101], position changes [102], running [21, 23], and carrying loads [22, 103, 104]. Considering level-ground walking, the spinal loading values we report closely match those in the literature, which adds confidence to the validity of our data and lends support to walking as an appropriate activity to normalize data to make comparisons. In this study, normalized L5/S1 FE, LB, and AR moments during walking were, respectively, 0.019, 0.016, and 0.007, similar to reports of 0.012, 0.009, and 0.007 [23] and 0.018, 0.026, and 0.007 in the literature [19]. Likewise, we observed normalized L5/S1 compression, AP shear, and ML shear forces of 1.25, 0.32, and 0.04, similar to reports of 1.17, 0.36, and 0.05 in the literature [23] and similar to reported normalized compression forces of 1.0–2.5 and peak AP shear forces of 0.6 during walking [19, 27, 105, 106].

Considering spinal loading during other activities reported in the literature, lifting a 27 kg weight from the ground produces a net L5/S1 FE moment of 450 Nm and compression force of 7,000 N [46]. Assuming a body mass of 70 kg and height of 1.78 m, this corresponds to normalized values of 0.37 for moment and 10.2 for force. In this study, a participant lifting a 20 kg plastic jug of water from the ground produced a (normalized) 0.21 net L5/S1 FE moment and 7.6 compressive force, which are similar values given the weight difference in loads. Likewise, carrying a 20 kg load in one hand while walking produces 2203 N of compression at L4/L5 [103], a normalized value of 3.21 assuming the same anthropometry as above. In this study, a participant carrying a 20 L plastic jug of water in one hand while walking produced a normalized net L4/L5 compressive force of 2.75.

Less data exist on occupational lumbar ES activity and spinal loading variability, although evidence suggests variability may be related to MSK health independent of magnitude. Performing constant repetitive motions over long durations is associated with higher risk of MSK conditions including back pain [85, 107, 108] and can lead to increased variability in spinal kinematics and kinetics during those repetitive activities over time [109]. Alternatively, varying spinal posture, hence varying the forces experienced by different structures in the spine, increases nutrient flow to intervertebral discs and reduces strain and creep on passive tissues of the spine such as the ligamentum flavum—factors thought beneficial to spine health [110–112]. Although no prior studies have explicitly tested differences in lumbar ES activity variability during occupational activities, individuals with back pain had reduced variability in lumbar ES activity during sustained sitting compared to healthy controls [113]. More research is needed to understand how varying back muscle activity and spinal loading during occupational activities affects physical characteristics related to back pain risk and the incidence of back pain. Moreover, the potential mitigating effects of leisure time PA on back muscle endurance warrant further study.

Although prior studies have compared back muscle endurance between groups [10, 11, 114], to our knowledge this is the first study to associate back muscle endurance with quantitative measures of occupational lumbar ES activity and spinal loading. The significant group difference in back muscle endurance we report (Fig 5) appears to be explained largely by individual differences in these magnitude and variability response variables. This is highlighted by the fact that we conducted partial correlations that accounted for body mass [79] as we found body mass to be negatively associated with back muscle endurance (*R* = -0.60, *P* = 0.031), which suggests higher back muscle endurance may be also partly attributable to having lower body mass. For example, after accounting for body mass, L5/S1 moment RMS explained over 80% of the variation in normalized slope of MF (*R* = 0.90), a very large effect according to Cohen’s criteria [80] (Fig 6). Similarly, after accounting for body mass, L5/S1 reaction force RMS explained more than 70% of the variation in normalized slope of MF (*R* = 0.85). Activating lumbar ES to more than twice walking value and to a higher mean magnitude each explained almost 50% of the variation in EMG-determined back muscle endurance with correlation coefficients greater than 0.6, a moderate-large effect. Subjecting L5/S1 to reaction forces greater than twice walking value during occupational work also explained close to 50% of the variation in EMG-determined back muscle endurance. Variability response variables for lumbar ES activity and L5/S1 moments and reaction forces also explained 50% of the variation in EMG-determined back endurance (Fig 7). These results suggest that loading the spine and activating the lumbar ES to higher magnitudes and with more variability at work may lead to the development of more fatigue-resistant back muscles.

The physical activity transition that motivated this study supports the mismatch hypothesis, that recent and intense changes in how people use their bodies–including reduced PA–have led to increases in the prevalence and severity of formerly rare conditions [115–118]. In particular, because humans are adapted to walk long distances often while carrying loads, as well as dig, climb and do other activities that require significant back muscle activity and spinal loading, the mismatch hypothesis predicts the human spine may be poorly adapted to experiencing only low levels of spinal loading and back muscle activation and thus developing weak back muscles with low endurance that are poor at stabilizing the spine, increasing risk of injury and back pain [7]. Back muscles are like guy wires providing the majority of support to the spine [119–121], so according to this reasoning features of the human spine thought to predispose humans to back pain, such as a longer lumbar region featuring a ventral curvature, known as lordosis [122–126], may only become problematic in the presence of weak, fatigable back muscles that are unable to generate appropriate levels of force to stabilize the spine [7].

The hypothesis that low back pain is a mismatch condition is supported by evidence that reduced back muscle endurance puts an individual at over three times higher risk for first-time back pain [8, 9], physically active subsistence farmers have higher back muscle endurance compared to more sedentary urbanites [10, 11], and moderate exercise is an effective treatment for back pain [12–14]. The findings presented here lend more partial support for the hypothesis that low back pain is a mismatch condition, as they provide additional evidence of higher back muscle endurance among physically active subsistence farmers and that back muscle endurance is strongly associated with the intensity and variability with which individuals load their spines and activate their back muscles during occupational work. Higher back muscle endurance may be preventive of low back pain for several reasons. Back muscle fatigue can reduce spinal stability [90, 127, 128], potentially lead to higher shear forces in the lumbar spine [129], reduce trunk neuromuscular control [130], delay muscle reaction times in response to a sudden load [131], increase muscle force variability [132, 133], and increase co-contraction of the abdominal muscles that could subject intervertebral joints to high loads [40, 134, 135], so more fatigue-resistant back muscles may, in theory, be less susceptible to these issues. Recent evidence that back pain prevalence may be high and rising in rural, nonindustrial regions [136, 137] suggests that, in regions undergoing urbanization and the physical activity transition, it is essential to promote occupational and leisure time PA that helps people develop and maintain high back muscle endurance.

### Limitations

There are several limitations to this study. First, as with other field-based studies [138] the intensive nature of data collection (e.g., 1 hr. focal follow with 10 wearable sensors) and remote sensor battery and on-device data storage requirements (e.g., no access to consistent electricity, 8 GB of IMU kinematic and EMG data generated per participant) necessarily limited the sample size. Second, data collection duration was limited to approximately 60 min of occupational work per participant on one day, and we did not measure leisure time PA such as sports nor time spent resting, which also affect overall spinal loading. We asked each participant to perform their most common, quotidian occupational activities while wearing the sensors, and survey data we collected on group differences in manual labor, walking, carrying, and housework habits give us confidence that the activities performed during sensor wear-time were valid representations of the typical work-related activities of the participants (Figs D-G in S2 File). As on-device sensor data storage and battery capabilities improve, future work should collect behavioral, IMU kinematic and EMG data continuously over days and weeks, within and between participants, and during different seasons. Third, because it was necessary to keep the sensors within 20 m of the receiving device, to troubleshoot potential issues with data recording in real time, and to document activities, we had to accompany participants during sensor wear-time and may therefore have introduced observation bias into the study. Fourth, while we did not use lower limb IMU or EMG sensors, lower limb contributions to spinal loads and back muscle activity are minimal because most loading results from stabilizing upper body mass above the pelvis [139, 140]. Fifth, the OpenSim model used a top-down modeling approach, and studies have shown low back loading, at least during gait, to be sensitive to top-down vs. bottom-up modeling approaches because bottom-up approaches are more sensitive to heel strike impulses [19, 20, 141]. Future work should compare these two approaches with this OpenSim model. Sixth, the OpenSim model did not include passive force contributions and we did not incorporate EMG optimization into the modeling approach. Net L5/S1 reaction forces can be sensitive to trunk muscle EMG, however the magnitude of these differences is ineffectually low (±4%) [142]. Finally, while the results support the hypotheses that experiencing greater loading magnitudes and variability during occupational work is associated with higher back muscle endurance, the cross-sectional study design does not directly address the source of higher back muscle endurance we observed for this rural subsistence population.

## Conclusions

We found higher magnitudes and increased variability of occupational back muscle activity and spinal loading to be positively associated with back muscle endurance among Rwandan subsistence farmers and office workers. Higher back muscle endurance may be also partly attributable to having lower body mass. These results suggest that the ongoing physical activity transition, in which machines are replacing human muscle for work and locomotion, likely has negative consequences for the human back and spine. Because back muscle endurance is the physical characteristic most strongly related to back pain risk, reduced intensity and variability of back muscle activity and spinal loading during occupational work may cause individuals to develop weaker, more fatigable backs susceptible to injury. There is a need for more cross-sectional studies on the effects of back muscle activity and spinal loading during occupational and leisure time activities on physical characteristics related to back pain risk. There is also a need for prospective studies on the incidence of back pain in populations that differ in subsistence strategy and PA level. As trends of urbanization continue and sedentary occupations become more common, we encourage global and public health officials to promote occupational and leisure time PA that helps people develop and maintain high back muscle endurance.

## Supporting information

S1 File Questionnaire on inclusivity in global research. (DOCX)

S2 File Supplemental figures and tables. (DOCX)

S3 File Microsoft Excel spreadsheet of model coefficient estimates and summary statistics. (XLSX)
