# Supplementary material for: Associations of back muscle endurance with occupational back muscle activity and spinal loading among subsistence farmers and office workers in Rwanda
Source: PLoS One. 2024 Nov 4;19(11):e0309658. doi: 10.1371/journal.pone.0309658 (PMC11534227; doi:10.1371/journal.pone.0309658)
Supplement: S2 File — (DOCX) [file pone.0309658.s002.docx]

**Supplemental figures and tables**

Associations of back muscle endurance with occupational back muscle activity and spinal loading among subsistence farmers and office workers in Rwanda

Benjamin E. Sibson^1^ *, Alexandra R. Harris^1^, Andrew K. Yegian^1^, Aimable Uwimana^2^, Assuman Nuhu^3^, Alec Thomas^4^, Dennis E. Anderson^5,6^, Robert Mang’eni Ojiambo^2^, Daniel E. Lieberman^1^

^1^ Department of Human Evolutionary Biology, Harvard University, Cambridge, MA, USA

^2^ Division of Basic Sciences, University of Global Health Equity, Butaro, RWA

^3^ Department of Physiotherapy, University of Rwanda, Kigali City, RWA

^4^ Institute of Sports Science, University of Lausanne, Lausanne, CH

^5^ Center for Advanced Orthopedic Studies, Beth Israel Deaconess Medical Center, Boston, MA, USA

^6^ Department of Orthopedic Surgery, Harvard Medical School, Boston, MA, USA

* Corresponding author

Email: [bsibson@g.harvard.edu](mailto:bsibson@g.harvard.edu) (BES)

**Fig A. Probability density functions of lumbar erector spinae activity proportions over twice walking value.**

>2 = proportion over twice walking; ES = erector spinae. Each subplot represents one participant. Value in parentheses are proportions over twice walking value, expressed as percentages. X-axis set to [0 10] for comparison.

**Fig B. Probability density functions of lumbosacral joint moment vector magnitude proportions over twice walking value.**

L5/S1 = lumbosacral joint; >2 = proportion over twice walking; VM = vector magnitude. Each subplot represents one participant. Value in parentheses are proportions over twice walking value, expressed as percentages. X-axis set to [0 0.2] for comparison.

**Fig C. Probability density functions of lumbosacral joint reaction force vector magnitude proportions over twice walking value.**

L5/S1 = lumbosacral joint; >2 = proportion over twice walking; VM = vector magnitude. Each subplot represents one participant. Value in parentheses are proportions over twice walking value, expressed as percentages. X-axis set to [0 5] for comparison.

**Fig D. Survey results for minutes per day of manual labor.**

Error bars are standard error of the mean. % diff = percentage difference between groups.

**Fig E. Survey results for minutes per day of walking at work.**

Error bars are standard error of the mean. % diff = percentage difference between groups.

**Fig F. Survey results for minutes per day of carrying.**

Error bars are standard error of the mean. % diff = percentage difference between groups.

**Fig G. Survey results for minutes per day of housework.**

Error bars are standard error of the mean. % diff = percentage difference between groups.

**Table A. General linear model coefficient estimates for lumbar erector spinae activity.**

| Response | Predictor | Coefficient Estimate | Standard Error | t-statistic | P-value |
| --- | --- | --- | --- | --- | --- |
| log(Mean) | (Intercept) | 0.301 | 0.797 | 0.377 | 0.715 |
|  | Age | 0.005 | 0.011 | 0.469 | 0.651 |
|  | Body Mass | 0.000 | 0.011 | -0.003 | 0.998 |
|  | Sex | 0.022 | 0.225 | 0.100 | 0.923 |
|  | Group | -1.082 | 0.262 | -4.136 | 0.003 |
| log(Proportion >2) | (Intercept) | 2.774 | 1.012 | 2.740 | 0.023 |
|  | Age | 0.006 | 0.014 | 0.392 | 0.705 |
|  | Body Mass | 0.005 | 0.014 | 0.353 | 0.732 |
|  | Sex | -0.129 | 0.285 | -0.451 | 0.663 |
|  | Group | -1.800 | 0.332 | -5.417 | 0.000 |
| log(IQR) | (Intercept) | 1.106 | 0.940 | 1.176 | 0.270 |
|  | Age | 0.003 | 0.013 | 0.201 | 0.846 |
|  | Body Mass | -0.011 | 0.013 | -0.806 | 0.441 |
|  | Sex | 0.008 | 0.265 | 0.029 | 0.978 |
|  | Group | -1.118 | 0.309 | -3.623 | 0.006 |
| log(SD) | (Intercept) | 0.427 | 0.714 | 0.598 | 0.564 |
|  | Age | -0.002 | 0.010 | -0.223 | 0.829 |
|  | Body Mass | 0.005 | 0.010 | 0.465 | 0.653 |
|  | Sex | 0.167 | 0.201 | 0.829 | 0.429 |
|  | Group | -1.004 | 0.234 | -4.287 | 0.002 |

>2 = proportion over twice walking; IQR = interquartile range; SD = standard deviation.

**Table B. General linear model summary statistics for lumbar erector spinae activity.**

| Response | Model | Sum of Squares | DF | Mean Squares | F-statistic | P-value |
| --- | --- | --- | --- | --- | --- | --- |
| log(Mean) | Total | 5.667 | 13 | 0.436 |  |  |
|  | Model | 4.234 | 4 | 1.059 | 6.649 | 0.009 |
|  | Residual | 1.433 | 9 | 0.159 |  |  |
| log(Proportion >2) | Total | 12.822 | 13 | 0.986 |  |  |
|  | Model | 10.511 | 4 | 2.628 | 10.235 | 0.002 |
|  | Residual | 2.311 | 9 | 0.257 |  |  |
| log(IQR) | Total | 7.530 | 13 | 0.579 |  |  |
|  | Model | 5.537 | 4 | 1.384 | 6.252 | 0.011 |
|  | Residual | 1.993 | 9 | 0.221 |  |  |
| log(SD) | Total | 4.524 | 13 | 0.348 |  |  |
|  | Model | 3.376 | 4 | 0.844 | 6.617 | 0.009 |
|  | Residual | 1.148 | 9 | 0.128 |  |  |

>2 = proportion over twice walking; IQR = interquartile range; SD = standard deviation.

**Table C. Descriptive statistics for net T1 – S1 joint moments.**

| Joint | Response | Axis | Rural Mean | Rural SD | Urban Mean | Urban SD | % diff |
| --- | --- | --- | --- | --- | --- | --- | --- |
| L5/S1 | RMS | VM | 0.048 | 0.009 | 0.029 | 0.007 | 68 |
|  |  | FE | 0.042 | 0.009 | 0.027 | 0.008 | 56 |
|  |  | LB | 0.019 | 0.003 | 0.008 | 0.002 | 146 |
|  |  | AR | 0.012 | 0.003 | 0.004 | 0.001 | 189 |
|  | Proportion >2 | VM | 24.107 | 9.484 | 8.617 | 12.171 | 180 |
|  |  | FE | 27.871 | 10.252 | 11.670 | 15.579 | 139 |
|  |  | LB | 8.291 | 5.773 | 1.179 | 0.983 | 603 |
|  |  | AR | 9.588 | 5.477 | 0.811 | 0.543 | 1083 |
|  | IQR | VM | 0.038 | 0.011 | 0.013 | 0.006 | 193 |
|  |  | FE | 0.036 | 0.012 | 0.015 | 0.007 | 141 |
|  |  | LB | 0.019 | 0.004 | 0.006 | 0.002 | 237 |
|  |  | AR | 0.010 | 0.002 | 0.003 | 0.001 | 263 |
|  | SD | VM | 0.031 | 0.006 | 0.012 | 0.005 | 152 |
|  |  | FE | 0.031 | 0.006 | 0.014 | 0.005 | 122 |
|  |  | LB | 0.019 | 0.002 | 0.007 | 0.001 | 161 |
|  |  | AR | 0.012 | 0.003 | 0.004 | 0.001 | 202 |
| L4/L5 | RMS | VM | 0.042 | 0.008 | 0.024 | 0.006 | 77 |
|  |  | FE | 0.037 | 0.008 | 0.022 | 0.007 | 64 |
|  |  | LB | 0.018 | 0.003 | 0.007 | 0.001 | 149 |
|  |  | AR | 0.010 | 0.003 | 0.003 | 0.001 | 213 |
|  | Proportion >2 | VM | 25.444 | 9.735 | 9.163 | 12.972 | 178 |
|  |  | FE | 28.826 | 10.751 | 13.685 | 17.126 | 111 |
|  |  | LB | 8.239 | 5.713 | 1.288 | 1.015 | 540 |
|  |  | AR | 9.024 | 4.441 | 0.689 | 0.443 | 1210 |
|  | IQR | VM | 0.033 | 0.010 | 0.011 | 0.005 | 194 |
|  |  | FE | 0.033 | 0.011 | 0.013 | 0.006 | 145 |
|  |  | LB | 0.017 | 0.003 | 0.005 | 0.001 | 237 |
|  |  | AR | 0.008 | 0.002 | 0.002 | 0.000 | 311 |
|  | SD | VM | 0.028 | 0.005 | 0.011 | 0.004 | 160 |
|  |  | FE | 0.029 | 0.006 | 0.012 | 0.004 | 129 |
|  |  | LB | 0.017 | 0.002 | 0.007 | 0.001 | 165 |
|  |  | AR | 0.010 | 0.003 | 0.003 | 0.001 | 220 |
| L3/L4 | RMS | VM | 0.038 | 0.008 | 0.021 | 0.006 | 79 |
|  |  | FE | 0.033 | 0.007 | 0.020 | 0.006 | 65 |
|  |  | LB | 0.016 | 0.002 | 0.006 | 0.001 | 154 |
|  |  | AR | 0.010 | 0.003 | 0.003 | 0.001 | 209 |
|  | Proportion >2 | VM | 25.457 | 9.703 | 9.521 | 13.915 | 167 |
|  |  | FE | 29.090 | 10.746 | 16.184 | 20.353 | 80 |
|  |  | LB | 9.067 | 5.981 | 1.417 | 1.044 | 540 |
|  |  | AR | 6.525 | 2.661 | 0.651 | 0.521 | 902 |
|  | IQR | VM | 0.029 | 0.009 | 0.010 | 0.004 | 191 |
|  |  | FE | 0.029 | 0.010 | 0.012 | 0.005 | 146 |
|  |  | LB | 0.015 | 0.003 | 0.005 | 0.001 | 223 |
|  |  | AR | 0.008 | 0.002 | 0.002 | 0.000 | 319 |
|  | SD | VM | 0.025 | 0.005 | 0.009 | 0.004 | 167 |
|  |  | FE | 0.026 | 0.006 | 0.011 | 0.004 | 135 |
|  |  | LB | 0.016 | 0.002 | 0.006 | 0.001 | 170 |
|  |  | AR | 0.010 | 0.002 | 0.003 | 0.001 | 217 |
| L2/L3 | RMS | VM | 0.034 | 0.007 | 0.020 | 0.005 | 74 |
|  |  | FE | 0.029 | 0.007 | 0.019 | 0.006 | 59 |
|  |  | LB | 0.015 | 0.002 | 0.006 | 0.001 | 159 |
|  |  | AR | 0.010 | 0.003 | 0.003 | 0.001 | 201 |
|  | Proportion >2 | VM | 26.364 | 10.533 | 9.749 | 14.557 | 170 |
|  |  | FE | 28.467 | 10.489 | 16.725 | 20.320 | 70 |
|  |  | LB | 9.124 | 5.703 | 1.662 | 1.323 | 449 |
|  |  | AR | 5.334 | 1.874 | 0.697 | 0.622 | 665 |
|  | IQR | VM | 0.026 | 0.008 | 0.009 | 0.004 | 182 |
|  |  | FE | 0.025 | 0.009 | 0.010 | 0.004 | 145 |
|  |  | LB | 0.013 | 0.003 | 0.004 | 0.001 | 212 |
|  |  | AR | 0.008 | 0.002 | 0.002 | 0.001 | 285 |
|  | SD | VM | 0.023 | 0.005 | 0.008 | 0.003 | 174 |
|  |  | FE | 0.023 | 0.005 | 0.009 | 0.003 | 142 |
|  |  | LB | 0.014 | 0.002 | 0.005 | 0.001 | 177 |
|  |  | AR | 0.010 | 0.002 | 0.003 | 0.000 | 209 |
| L1/L2 | RMS | VM | 0.032 | 0.007 | 0.019 | 0.005 | 64 |
|  |  | FE | 0.027 | 0.006 | 0.018 | 0.005 | 49 |
|  |  | LB | 0.013 | 0.002 | 0.005 | 0.001 | 166 |
|  |  | AR | 0.010 | 0.002 | 0.003 | 0.001 | 191 |
|  | Proportion >2 | VM | 25.914 | 10.925 | 9.709 | 15.351 | 167 |
|  |  | FE | 27.453 | 10.271 | 16.481 | 20.123 | 67 |
|  |  | LB | 9.311 | 4.936 | 1.689 | 1.381 | 451 |
|  |  | AR | 4.809 | 1.625 | 0.753 | 0.827 | 539 |
|  | IQR | VM | 0.023 | 0.007 | 0.008 | 0.003 | 180 |
|  |  | FE | 0.021 | 0.007 | 0.009 | 0.003 | 143 |
|  |  | LB | 0.011 | 0.002 | 0.004 | 0.001 | 196 |
|  |  | AR | 0.008 | 0.002 | 0.002 | 0.001 | 250 |
|  | SD | VM | 0.021 | 0.005 | 0.007 | 0.002 | 180 |
|  |  | FE | 0.020 | 0.005 | 0.008 | 0.002 | 150 |
|  |  | LB | 0.013 | 0.002 | 0.004 | 0.001 | 185 |
|  |  | AR | 0.010 | 0.002 | 0.003 | 0.001 | 201 |
| T12/L1 | RMS | VM | 0.030 | 0.007 | 0.019 | 0.004 | 52 |
|  |  | FE | 0.025 | 0.006 | 0.019 | 0.004 | 37 |
|  |  | LB | 0.012 | 0.002 | 0.004 | 0.000 | 168 |
|  |  | AR | 0.009 | 0.003 | 0.003 | 0.001 | 199 |
|  | Proportion >2 | VM | 25.137 | 10.318 | 8.866 | 15.430 | 184 |
|  |  | FE | 25.645 | 10.963 | 13.830 | 19.518 | 85 |
|  |  | LB | 10.319 | 5.922 | 1.857 | 1.450 | 456 |
|  |  | AR | 4.959 | 1.615 | 0.821 | 0.903 | 504 |
|  | IQR | VM | 0.020 | 0.006 | 0.007 | 0.003 | 167 |
|  |  | FE | 0.018 | 0.006 | 0.007 | 0.003 | 138 |
|  |  | LB | 0.010 | 0.002 | 0.004 | 0.001 | 174 |
|  |  | AR | 0.008 | 0.002 | 0.002 | 0.001 | 238 |
|  | SD | VM | 0.019 | 0.005 | 0.007 | 0.002 | 188 |
|  |  | FE | 0.018 | 0.005 | 0.007 | 0.002 | 160 |
|  |  | LB | 0.012 | 0.002 | 0.004 | 0.001 | 189 |
|  |  | AR | 0.009 | 0.002 | 0.003 | 0.000 | 210 |
| T11/T12 | RMS | VM | 0.028 | 0.006 | 0.020 | 0.004 | 42 |
|  |  | FE | 0.024 | 0.006 | 0.019 | 0.004 | 27 |
|  |  | LB | 0.011 | 0.002 | 0.004 | 0.000 | 167 |
|  |  | AR | 0.009 | 0.003 | 0.003 | 0.001 | 214 |
|  | Proportion >2 | VM | 23.305 | 10.571 | 7.131 | 12.983 | 227 |
|  |  | FE | 23.270 | 11.032 | 11.046 | 17.546 | 111 |
|  |  | LB | 10.604 | 5.292 | 2.822 | 2.219 | 276 |
|  |  | AR | 5.388 | 1.766 | 0.784 | 0.869 | 587 |
|  | IQR | VM | 0.017 | 0.005 | 0.006 | 0.002 | 162 |
|  |  | FE | 0.015 | 0.005 | 0.006 | 0.002 | 134 |
|  |  | LB | 0.009 | 0.002 | 0.003 | 0.001 | 149 |
|  |  | AR | 0.007 | 0.002 | 0.002 | 0.001 | 230 |
|  | SD | VM | 0.017 | 0.005 | 0.006 | 0.001 | 199 |
|  |  | FE | 0.016 | 0.005 | 0.006 | 0.001 | 175 |
|  |  | LB | 0.011 | 0.002 | 0.004 | 0.001 | 191 |
|  |  | AR | 0.009 | 0.003 | 0.003 | 0.000 | 225 |
| T10/T11 | RMS | VM | 0.026 | 0.006 | 0.019 | 0.003 | 36 |
|  |  | FE | 0.023 | 0.005 | 0.019 | 0.003 | 21 |
|  |  | LB | 0.010 | 0.002 | 0.004 | 0.000 | 165 |
|  |  | AR | 0.009 | 0.003 | 0.003 | 0.001 | 236 |
|  | Proportion >2 | VM | 20.776 | 10.286 | 5.882 | 12.110 | 253 |
|  |  | FE | 19.922 | 11.222 | 8.207 | 14.796 | 143 |
|  |  | LB | 11.353 | 5.634 | 2.917 | 2.301 | 289 |
|  |  | AR | 6.222 | 2.149 | 0.849 | 1.015 | 633 |
|  | IQR | VM | 0.015 | 0.005 | 0.006 | 0.002 | 161 |
|  |  | FE | 0.012 | 0.004 | 0.005 | 0.001 | 127 |
|  |  | LB | 0.008 | 0.002 | 0.003 | 0.001 | 131 |
|  |  | AR | 0.006 | 0.002 | 0.002 | 0.001 | 219 |
|  | SD | VM | 0.016 | 0.005 | 0.005 | 0.001 | 214 |
|  |  | FE | 0.015 | 0.006 | 0.005 | 0.001 | 191 |
|  |  | LB | 0.010 | 0.002 | 0.003 | 0.001 | 190 |
|  |  | AR | 0.008 | 0.003 | 0.002 | 0.000 | 247 |
| T9/T10 | RMS | VM | 0.025 | 0.006 | 0.019 | 0.003 | 31 |
|  |  | FE | 0.021 | 0.005 | 0.018 | 0.003 | 16 |
|  |  | LB | 0.009 | 0.002 | 0.004 | 0.000 | 163 |
|  |  | AR | 0.008 | 0.003 | 0.002 | 0.000 | 258 |
|  | Proportion >2 | VM | 18.629 | 10.892 | 5.071 | 11.299 | 267 |
|  |  | FE | 16.892 | 10.630 | 7.117 | 14.287 | 137 |
|  |  | LB | 11.382 | 5.082 | 3.023 | 2.419 | 277 |
|  |  | AR | 6.486 | 2.184 | 0.775 | 0.898 | 737 |
|  | IQR | VM | 0.012 | 0.004 | 0.005 | 0.001 | 157 |
|  |  | FE | 0.010 | 0.003 | 0.005 | 0.001 | 111 |
|  |  | LB | 0.007 | 0.002 | 0.003 | 0.001 | 111 |
|  |  | AR | 0.006 | 0.002 | 0.002 | 0.001 | 209 |
|  | SD | VM | 0.015 | 0.005 | 0.004 | 0.001 | 234 |
|  |  | FE | 0.014 | 0.006 | 0.005 | 0.001 | 211 |
|  |  | LB | 0.009 | 0.002 | 0.003 | 0.001 | 190 |
|  |  | AR | 0.008 | 0.003 | 0.002 | 0.000 | 269 |
| T8/T9 | RMS | VM | 0.023 | 0.006 | 0.018 | 0.002 | 29 |
|  |  | FE | 0.020 | 0.005 | 0.018 | 0.002 | 13 |
|  |  | LB | 0.009 | 0.002 | 0.003 | 0.000 | 160 |
|  |  | AR | 0.008 | 0.003 | 0.002 | 0.000 | 283 |
|  | Proportion >2 | VM | 17.197 | 10.578 | 4.341 | 10.050 | 296 |
|  |  | FE | 14.105 | 9.423 | 6.325 | 13.243 | 123 |
|  |  | LB | 12.017 | 5.490 | 2.986 | 2.450 | 302 |
|  |  | AR | 7.363 | 2.681 | 0.773 | 0.936 | 853 |
|  | IQR | VM | 0.011 | 0.004 | 0.004 | 0.001 | 141 |
|  |  | FE | 0.008 | 0.003 | 0.005 | 0.001 | 83 |
|  |  | LB | 0.006 | 0.002 | 0.003 | 0.001 | 95 |
|  |  | AR | 0.005 | 0.002 | 0.002 | 0.001 | 201 |
|  | SD | VM | 0.014 | 0.006 | 0.004 | 0.001 | 252 |
|  |  | FE | 0.013 | 0.006 | 0.004 | 0.001 | 229 |
|  |  | LB | 0.009 | 0.002 | 0.003 | 0.000 | 188 |
|  |  | AR | 0.008 | 0.003 | 0.002 | 0.000 | 294 |
| T7/T8 | RMS | VM | 0.022 | 0.006 | 0.017 | 0.002 | 27 |
|  |  | FE | 0.019 | 0.005 | 0.017 | 0.002 | 11 |
|  |  | LB | 0.008 | 0.002 | 0.003 | 0.000 | 156 |
|  |  | AR | 0.008 | 0.003 | 0.002 | 0.000 | 308 |
|  | Proportion >2 | VM | 16.321 | 10.140 | 5.031 | 11.674 | 224 |
|  |  | FE | 12.988 | 9.374 | 6.569 | 13.726 | 98 |
|  |  | LB | 12.241 | 5.692 | 3.102 | 2.426 | 295 |
|  |  | AR | 8.239 | 3.384 | 0.761 | 0.940 | 983 |
|  | IQR | VM | 0.009 | 0.004 | 0.004 | 0.001 | 120 |
|  |  | FE | 0.007 | 0.003 | 0.004 | 0.001 | 65 |
|  |  | LB | 0.005 | 0.001 | 0.003 | 0.001 | 81 |
|  |  | AR | 0.005 | 0.002 | 0.002 | 0.001 | 187 |
|  | SD | VM | 0.013 | 0.006 | 0.004 | 0.001 | 269 |
|  |  | FE | 0.013 | 0.006 | 0.004 | 0.001 | 246 |
|  |  | LB | 0.008 | 0.002 | 0.003 | 0.000 | 185 |
|  |  | AR | 0.008 | 0.003 | 0.002 | 0.000 | 320 |
| T6/T7 | RMS | VM | 0.020 | 0.006 | 0.016 | 0.002 | 28 |
|  |  | FE | 0.017 | 0.005 | 0.016 | 0.002 | 10 |
|  |  | LB | 0.007 | 0.002 | 0.003 | 0.000 | 153 |
|  |  | AR | 0.008 | 0.004 | 0.002 | 0.000 | 330 |
|  | Proportion >2 | VM | 16.817 | 10.688 | 5.581 | 12.719 | 201 |
|  |  | FE | 12.026 | 8.619 | 7.866 | 14.456 | 53 |
|  |  | LB | 13.536 | 6.113 | 3.288 | 2.482 | 312 |
|  |  | AR | 8.677 | 3.096 | 0.698 | 0.853 | 1142 |
|  | IQR | VM | 0.008 | 0.004 | 0.004 | 0.001 | 106 |
|  |  | FE | 0.006 | 0.002 | 0.004 | 0.001 | 49 |
|  |  | LB | 0.005 | 0.001 | 0.003 | 0.001 | 67 |
|  |  | AR | 0.005 | 0.002 | 0.002 | 0.001 | 165 |
|  | SD | VM | 0.013 | 0.006 | 0.003 | 0.001 | 281 |
|  |  | FE | 0.012 | 0.006 | 0.003 | 0.001 | 258 |
|  |  | LB | 0.007 | 0.002 | 0.003 | 0.000 | 183 |
|  |  | AR | 0.008 | 0.004 | 0.002 | 0.000 | 344 |
| T5/T6 | RMS | VM | 0.019 | 0.006 | 0.015 | 0.002 | 30 |
|  |  | FE | 0.016 | 0.005 | 0.014 | 0.002 | 11 |
|  |  | LB | 0.007 | 0.002 | 0.003 | 0.000 | 152 |
|  |  | AR | 0.008 | 0.004 | 0.002 | 0.000 | 342 |
|  | Proportion >2 | VM | 17.713 | 12.219 | 7.402 | 15.496 | 139 |
|  |  | FE | 11.625 | 8.088 | 15.172 | 18.821 | -23 |
|  |  | LB | 13.227 | 5.446 | 3.383 | 2.509 | 291 |
|  |  | AR | 9.067 | 2.904 | 0.681 | 0.811 | 1231 |
|  | IQR | VM | 0.008 | 0.004 | 0.004 | 0.001 | 98 |
|  |  | FE | 0.005 | 0.002 | 0.004 | 0.001 | 38 |
|  |  | LB | 0.004 | 0.001 | 0.003 | 0.001 | 57 |
|  |  | AR | 0.004 | 0.002 | 0.002 | 0.001 | 144 |
|  | SD | VM | 0.013 | 0.006 | 0.003 | 0.001 | 286 |
|  |  | FE | 0.012 | 0.006 | 0.003 | 0.001 | 261 |
|  |  | LB | 0.007 | 0.002 | 0.002 | 0.000 | 182 |
|  |  | AR | 0.008 | 0.004 | 0.002 | 0.000 | 361 |
| T4/T5 | RMS | VM | 0.018 | 0.007 | 0.013 | 0.002 | 35 |
|  |  | FE | 0.014 | 0.005 | 0.013 | 0.002 | 13 |
|  |  | LB | 0.006 | 0.002 | 0.002 | 0.000 | 153 |
|  |  | AR | 0.008 | 0.004 | 0.002 | 0.000 | 343 |
|  | Proportion >2 | VM | 18.634 | 12.207 | 13.692 | 19.202 | 36 |
|  |  | FE | 12.772 | 9.776 | 27.515 | 28.056 | -54 |
|  |  | LB | 13.128 | 5.266 | 3.254 | 2.427 | 303 |
|  |  | AR | 9.188 | 3.226 | 0.693 | 0.747 | 1226 |
|  | IQR | VM | 0.007 | 0.004 | 0.004 | 0.001 | 99 |
|  |  | FE | 0.005 | 0.002 | 0.004 | 0.001 | 36 |
|  |  | LB | 0.004 | 0.001 | 0.002 | 0.001 | 49 |
|  |  | AR | 0.004 | 0.002 | 0.002 | 0.001 | 123 |
|  | SD | VM | 0.013 | 0.006 | 0.003 | 0.001 | 286 |
|  |  | FE | 0.012 | 0.006 | 0.003 | 0.001 | 256 |
|  |  | LB | 0.006 | 0.002 | 0.002 | 0.000 | 185 |
|  |  | AR | 0.008 | 0.004 | 0.002 | 0.000 | 367 |
| T3/T4 | RMS | VM | 0.017 | 0.007 | 0.012 | 0.002 | 44 |
|  |  | FE | 0.013 | 0.005 | 0.011 | 0.002 | 19 |
|  |  | LB | 0.006 | 0.002 | 0.002 | 0.000 | 159 |
|  |  | AR | 0.008 | 0.004 | 0.002 | 0.000 | 340 |
|  | Proportion >2 | VM | 20.293 | 12.804 | 22.212 | 23.489 | -9 |
|  |  | FE | 13.762 | 9.751 | 35.585 | 30.681 | -61 |
|  |  | LB | 12.573 | 5.133 | 3.134 | 2.551 | 301 |
|  |  | AR | 10.029 | 2.893 | 0.732 | 0.688 | 1269 |
|  | IQR | VM | 0.007 | 0.004 | 0.004 | 0.001 | 100 |
|  |  | FE | 0.005 | 0.002 | 0.004 | 0.001 | 37 |
|  |  | LB | 0.003 | 0.001 | 0.002 | 0.001 | 44 |
|  |  | AR | 0.004 | 0.002 | 0.002 | 0.001 | 110 |
|  | SD | VM | 0.012 | 0.006 | 0.003 | 0.001 | 287 |
|  |  | FE | 0.012 | 0.006 | 0.003 | 0.001 | 246 |
|  |  | LB | 0.006 | 0.002 | 0.002 | 0.000 | 191 |
|  |  | AR | 0.008 | 0.004 | 0.002 | 0.000 | 364 |
| T2/T3 | RMS | VM | 0.016 | 0.007 | 0.010 | 0.002 | 55 |
|  |  | FE | 0.013 | 0.006 | 0.010 | 0.002 | 27 |
|  |  | LB | 0.006 | 0.002 | 0.002 | 0.000 | 166 |
|  |  | AR | 0.008 | 0.004 | 0.002 | 0.000 | 333 |
|  | Proportion >2 | VM | 22.207 | 14.413 | 30.287 | 25.736 | -27 |
|  |  | FE | 13.973 | 9.015 | 50.172 | 30.626 | -72 |
|  |  | LB | 11.277 | 4.972 | 2.963 | 2.709 | 281 |
|  |  | AR | 9.860 | 2.885 | 0.917 | 0.730 | 975 |
|  | IQR | VM | 0.008 | 0.005 | 0.004 | 0.001 | 101 |
|  |  | FE | 0.006 | 0.002 | 0.004 | 0.001 | 37 |
|  |  | LB | 0.003 | 0.001 | 0.002 | 0.001 | 45 |
|  |  | AR | 0.004 | 0.002 | 0.002 | 0.001 | 101 |
|  | SD | VM | 0.012 | 0.006 | 0.003 | 0.001 | 295 |
|  |  | FE | 0.012 | 0.006 | 0.004 | 0.001 | 236 |
|  |  | LB | 0.006 | 0.002 | 0.002 | 0.000 | 199 |
|  |  | AR | 0.008 | 0.004 | 0.002 | 0.000 | 358 |
| T1/T2 | RMS | VM | 0.016 | 0.007 | 0.009 | 0.002 | 69 |
|  |  | FE | 0.012 | 0.006 | 0.009 | 0.002 | 38 |
|  |  | LB | 0.006 | 0.001 | 0.002 | 0.000 | 174 |
|  |  | AR | 0.008 | 0.004 | 0.002 | 0.000 | 329 |
|  | Proportion >2 | VM | 22.279 | 13.340 | 33.281 | 21.631 | -33 |
|  |  | FE | 13.575 | 7.799 | 56.658 | 27.913 | -76 |
|  |  | LB | 10.444 | 4.981 | 1.816 | 2.180 | 475 |
|  |  | AR | 9.956 | 3.357 | 1.078 | 0.918 | 823 |
|  | IQR | VM | 0.008 | 0.005 | 0.004 | 0.001 | 105 |
|  |  | FE | 0.006 | 0.002 | 0.004 | 0.001 | 39 |
|  |  | LB | 0.003 | 0.001 | 0.002 | 0.001 | 50 |
|  |  | AR | 0.004 | 0.002 | 0.002 | 0.001 | 97 |
|  | SD | VM | 0.012 | 0.006 | 0.003 | 0.001 | 310 |
|  |  | FE | 0.012 | 0.006 | 0.004 | 0.001 | 227 |
|  |  | LB | 0.006 | 0.002 | 0.002 | 0.000 | 206 |
|  |  | AR | 0.008 | 0.004 | 0.002 | 0.000 | 353 |

RMS = root mean square; >2 = proportion over twice walking; IQR = interquartile range; SD = standard deviation; VM = vector magnitude; FE = flexion-extension; LB = lateral bending; AR = axial rotation.

**Table D. General linear model coefficient estimates for net lumbosacral joint moments.**

| Axis | Response | Predictor | Coefficient Estimate | Standard Error | t-statistic | P-value |
| --- | --- | --- | --- | --- | --- | --- |
| VM | log(RMS) | (Intercept) | -2.828 | 0.474 | -5.965 | 0.000 |
|  |  | Age | 0.005 | 0.007 | 0.703 | 0.500 |
|  |  | Body Mass | -0.006 | 0.007 | -0.920 | 0.382 |
|  |  | Sex | -0.045 | 0.133 | -0.337 | 0.744 |
|  |  | Group | -0.450 | 0.156 | -2.892 | 0.018 |
|  | log(Proportion >2) | (Intercept) | 3.850 | 1.936 | 1.989 | 0.078 |
|  |  | Age | 0.051 | 0.028 | 1.847 | 0.098 |
|  |  | Body Mass | -0.036 | 0.027 | -1.317 | 0.221 |
|  |  | Sex | -0.895 | 0.545 | -1.642 | 0.135 |
|  |  | Group | -1.601 | 0.636 | -2.519 | 0.033 |
|  | log(IQR) | (Intercept) | -3.137 | 0.858 | -3.656 | 0.005 |
|  |  | Age | 0.008 | 0.012 | 0.664 | 0.523 |
|  |  | Body Mass | -0.007 | 0.012 | -0.557 | 0.591 |
|  |  | Sex | -0.145 | 0.242 | -0.601 | 0.563 |
|  |  | Group | -1.047 | 0.282 | -3.717 | 0.005 |
|  | log(SD) | (Intercept) | -3.432 | 0.546 | -6.286 | 0.000 |
|  |  | Age | 0.012 | 0.008 | 1.535 | 0.159 |
|  |  | Body Mass | -0.007 | 0.008 | -0.973 | 0.356 |
|  |  | Sex | -0.114 | 0.154 | -0.743 | 0.476 |
|  |  | Group | -0.865 | 0.179 | -4.826 | 0.001 |
| FE | log(RMS) | (Intercept) | -2.961 | 0.573 | -5.163 | 0.001 |
|  |  | Age | 0.005 | 0.008 | 0.669 | 0.520 |
|  |  | Body Mass | -0.006 | 0.008 | -0.786 | 0.452 |
|  |  | Sex | -0.079 | 0.161 | -0.486 | 0.638 |
|  |  | Group | -0.390 | 0.188 | -2.071 | 0.068 |
|  | log(Proportion >2) | (Intercept) | 1.657 | 1.587 | 1.044 | 0.324 |
|  |  | Age | 0.045 | 0.023 | 1.967 | 0.081 |
|  |  | Body Mass | 0.005 | 0.022 | 0.216 | 0.833 |
|  |  | Sex | -0.620 | 0.447 | -1.388 | 0.199 |
|  |  | Group | -1.525 | 0.521 | -2.928 | 0.017 |
|  | log(IQR) | (Intercept) | -2.838 | 0.966 | -2.938 | 0.017 |
|  |  | Age | 0.000 | 0.014 | -0.019 | 0.986 |
|  |  | Body Mass | -0.008 | 0.014 | -0.606 | 0.559 |
|  |  | Sex | -0.037 | 0.272 | -0.138 | 0.894 |
|  |  | Group | -0.849 | 0.317 | -2.679 | 0.025 |
|  | log(SD) | (Intercept) | -3.068 | 0.570 | -5.386 | 0.000 |
|  |  | Age | 0.007 | 0.008 | 0.811 | 0.438 |
|  |  | Body Mass | -0.011 | 0.008 | -1.343 | 0.212 |
|  |  | Sex | -0.026 | 0.160 | -0.159 | 0.877 |
|  |  | Group | -0.692 | 0.187 | -3.701 | 0.005 |
| LB | log(RMS) | (Intercept) | -3.763 | 0.382 | -9.859 | 0.000 |
|  |  | Age | -0.001 | 0.005 | -0.266 | 0.796 |
|  |  | Body Mass | -0.003 | 0.005 | -0.580 | 0.576 |
|  |  | Sex | 0.113 | 0.107 | 1.054 | 0.319 |
|  |  | Group | -0.860 | 0.125 | -6.867 | 0.000 |
|  | log(Proportion >2) | (Intercept) | 6.310 | 1.484 | 4.251 | 0.002 |
|  |  | Age | 0.026 | 0.021 | 1.215 | 0.255 |
|  |  | Body Mass | -0.080 | 0.021 | -3.855 | 0.004 |
|  |  | Sex | -0.922 | 0.418 | -2.206 | 0.055 |
|  |  | Group | -1.637 | 0.487 | -3.360 | 0.008 |
|  | log(IQR) | (Intercept) | -3.388 | 0.590 | -5.741 | 0.000 |
|  |  | Age | 0.000 | 0.008 | 0.010 | 0.993 |
|  |  | Body Mass | -0.010 | 0.008 | -1.176 | 0.270 |
|  |  | Sex | -0.030 | 0.166 | -0.183 | 0.859 |
|  |  | Group | -1.138 | 0.194 | -5.874 | 0.000 |
|  | log(SD) | (Intercept) | -4.054 | 0.282 | -14.354 | 0.000 |
|  |  | Age | 0.003 | 0.004 | 0.640 | 0.538 |
|  |  | Body Mass | -0.001 | 0.004 | -0.285 | 0.782 |
|  |  | Sex | 0.076 | 0.080 | 0.955 | 0.364 |
|  |  | Group | -0.926 | 0.093 | -9.990 | 0.000 |
| AR | log(RMS) | (Intercept) | -4.138 | 0.506 | -8.173 | 0.000 |
|  |  | Age | 0.001 | 0.007 | 0.165 | 0.873 |
|  |  | Body Mass | -0.006 | 0.007 | -0.871 | 0.406 |
|  |  | Sex | 0.051 | 0.143 | 0.359 | 0.728 |
|  |  | Group | -0.969 | 0.166 | -5.832 | 0.000 |
|  | log(Proportion >2) | (Intercept) | 3.755 | 1.867 | 2.011 | 0.075 |
|  |  | Age | 0.012 | 0.027 | 0.459 | 0.657 |
|  |  | Body Mass | -0.031 | 0.026 | -1.192 | 0.264 |
|  |  | Sex | -0.500 | 0.526 | -0.951 | 0.366 |
|  |  | Group | -2.336 | 0.613 | -3.812 | 0.004 |
|  | log(IQR) | (Intercept) | -4.896 | 0.554 | -8.839 | 0.000 |
|  |  | Age | 0.011 | 0.008 | 1.393 | 0.197 |
|  |  | Body Mass | -0.001 | 0.008 | -0.155 | 0.881 |
|  |  | Sex | -0.100 | 0.156 | -0.639 | 0.539 |
|  |  | Group | -1.284 | 0.182 | -7.065 | 0.000 |
|  | log(SD) | (Intercept) | -4.365 | 0.531 | -8.214 | 0.000 |
|  |  | Age | 0.004 | 0.008 | 0.480 | 0.643 |
|  |  | Body Mass | -0.004 | 0.007 | -0.535 | 0.605 |
|  |  | Sex | 0.046 | 0.150 | 0.306 | 0.767 |
|  |  | Group | -1.032 | 0.174 | -5.914 | 0.000 |

RMS = root mean square; >2 = proportion over twice walking; IQR = interquartile range; SD = standard deviation; VM = vector magnitude; FE = flexion-extension; LB = lateral bending; AR = axial rotation.

**Table E. General linear model summary statistics for net lumbosacral joint moments.**

| Axis | Response | Model | Sum of Squares | DF | Mean Squares | F | P |
| --- | --- | --- | --- | --- | --- | --- | --- |
| VM | log(RMS) | Total | 1.567 | 13 | 0.121 |  |  |
|  |  | Model | 1.061 | 4 | 0.265 | 4.711 | 0.025 |
|  |  | Residual | 0.507 | 9 | 0.056 |  |  |
|  | log(Proportion >2) | Total | 31.562 | 13 | 2.428 |  |  |
|  |  | Model | 23.111 | 4 | 5.778 | 6.153 | 0.011 |
|  |  | Residual | 8.451 | 9 | 0.939 |  |  |
|  | log(IQR) | Total | 6.286 | 13 | 0.484 |  |  |
|  |  | Model | 4.626 | 4 | 1.157 | 6.271 | 0.011 |
|  |  | Residual | 1.66 | 9 | 0.184 |  |  |
|  | log(SD) | Total | 4.264 | 13 | 0.328 |  |  |
|  |  | Model | 3.592 | 4 | 0.898 | 12.03 | 0.001 |
|  |  | Residual | 0.672 | 9 | 0.075 |  |  |
| FE | log(RMS) | Total | 1.627 | 13 | 0.125 |  |  |
|  |  | Model | 0.886 | 4 | 0.221 | 2.688 | 0.1 |
|  |  | Residual | 0.741 | 9 | 0.082 |  |  |
|  | log(Proportion >2) | Total | 17.396 | 13 | 1.338 |  |  |
|  |  | Model | 11.72 | 4 | 2.93 | 4.646 | 0.026 |
|  |  | Residual | 5.676 | 9 | 0.631 |  |  |
|  | log(IQR) | Total | 5.218 | 13 | 0.401 |  |  |
|  |  | Model | 3.115 | 4 | 0.779 | 3.333 | 0.062 |
|  |  | Residual | 2.103 | 9 | 0.234 |  |  |
|  | log(SD) | Total | 3.351 | 13 | 0.258 |  |  |
|  |  | Model | 2.62 | 4 | 0.655 | 8.058 | 0.005 |
|  |  | Residual | 0.731 | 9 | 0.081 |  |  |
| LB | log(RMS) | Total | 3.213 | 13 | 0.247 |  |  |
|  |  | Model | 2.884 | 4 | 0.721 | 19.764 | 0 |
|  |  | Residual | 0.328 | 9 | 0.036 |  |  |
|  | log(Proportion >2) | Total | 41.123 | 13 | 3.163 |  |  |
|  |  | Model | 36.158 | 4 | 9.039 | 16.386 | 0 |
|  |  | Residual | 4.965 | 9 | 0.552 |  |  |
|  | log(IQR) | Total | 6.24 | 13 | 0.48 |  |  |
|  |  | Model | 5.455 | 4 | 1.364 | 15.642 | 0 |
|  |  | Residual | 0.785 | 9 | 0.087 |  |  |
|  | log(SD) | Total | 3.376 | 13 | 0.26 |  |  |
|  |  | Model | 3.196 | 4 | 0.799 | 39.993 | 0 |
|  |  | Residual | 0.18 | 9 | 0.02 |  |  |
| AR | log(RMS) | Total | 4.436 | 13 | 0.341 |  |  |
|  |  | Model | 3.859 | 4 | 0.965 | 15.027 | 0.001 |
|  |  | Residual | 0.578 | 9 | 0.064 |  |  |
|  | log(Proportion >2) | Total | 34.921 | 13 | 2.686 |  |  |
|  |  | Model | 27.064 | 4 | 6.766 | 7.75 | 0.005 |
|  |  | Residual | 7.857 | 9 | 0.873 |  |  |
|  | log(IQR) | Total | 6.881 | 13 | 0.529 |  |  |
|  |  | Model | 6.189 | 4 | 1.547 | 20.137 | 0 |
|  |  | Residual | 0.692 | 9 | 0.077 |  |  |
|  | log(SD) | Total | 4.817 | 13 | 0.371 |  |  |
|  |  | Model | 4.181 | 4 | 1.045 | 14.774 | 0.001 |
|  |  | Residual | 0.637 | 9 | 0.071 |  |  |

RMS = root mean square; >2 = proportion over twice walking; IQR = interquartile range; SD = standard deviation; VM = vector magnitude; FE = flexion-extension; LB = lateral bending; AR = axial rotation.

**Table F. Descriptive statistics for net T1 – S1 joint reaction forces.**

| Joint | Response | Axis | Rural Mean | Rural SD | Urban Mean | Urban SD | % diff |
| --- | --- | --- | --- | --- | --- | --- | --- |
| L5/S1 | RMS | VM | 1.878 | 0.241 | 1.228 | 0.113 | 53 |
|  |  | C | 1.813 | 0.228 | 1.192 | 0.111 | 52 |
|  |  | AP | 0.481 | 0.088 | 0.293 | 0.034 | 64 |
|  |  | ML | 0.071 | 0.015 | 0.035 | 0.008 | 103 |
|  | Proportion >2 | VM | 12.400 | 5.408 | 0.826 | 0.852 | 1401 |
|  |  | C | 12.186 | 4.697 | 0.787 | 0.807 | 1448 |
|  |  | AP | 14.323 | 6.013 | 1.156 | 1.342 | 1139 |
|  |  | ML | 6.169 | 4.224 | 1.389 | 1.199 | 344 |
|  | IQR | VM | 1.020 | 0.203 | 0.323 | 0.133 | 216 |
|  |  | C | 0.984 | 0.192 | 0.312 | 0.130 | 216 |
|  |  | AP | 0.266 | 0.070 | 0.083 | 0.031 | 220 |
|  |  | ML | 0.059 | 0.015 | 0.030 | 0.005 | 94 |
|  | SD | VM | 0.891 | 0.155 | 0.344 | 0.079 | 159 |
|  |  | C | 0.857 | 0.145 | 0.331 | 0.078 | 159 |
|  |  | AP | 0.240 | 0.059 | 0.095 | 0.019 | 154 |
|  |  | ML | 0.069 | 0.016 | 0.032 | 0.009 | 114 |
| L4/L5 | RMS | VM | 1.423 | 0.178 | 0.928 | 0.095 | 53 |
|  |  | C | 1.412 | 0.176 | 0.924 | 0.094 | 53 |
|  |  | AP | 0.176 | 0.031 | 0.088 | 0.013 | 100 |
|  |  | ML | 0.040 | 0.009 | 0.020 | 0.005 | 101 |
|  | Proportion >2 | VM | 13.604 | 5.542 | 0.883 | 0.962 | 1440 |
|  |  | C | 13.168 | 5.431 | 0.875 | 0.901 | 1405 |
|  |  | AP | 23.387 | 7.891 | 2.577 | 3.417 | 807 |
|  |  | ML | 7.420 | 5.667 | 1.490 | 1.294 | 398 |
|  | IQR | VM | 0.790 | 0.152 | 0.250 | 0.106 | 217 |
|  |  | C | 0.783 | 0.149 | 0.248 | 0.105 | 216 |
|  |  | AP | 0.109 | 0.029 | 0.037 | 0.016 | 195 |
|  |  | ML | 0.033 | 0.009 | 0.017 | 0.004 | 100 |
|  | SD | VM | 0.681 | 0.110 | 0.260 | 0.067 | 162 |
|  |  | C | 0.673 | 0.108 | 0.257 | 0.066 | 161 |
|  |  | AP | 0.106 | 0.023 | 0.041 | 0.009 | 160 |
|  |  | ML | 0.039 | 0.009 | 0.018 | 0.006 | 113 |
| L3/L4 | RMS | VM | 1.266 | 0.155 | 0.830 | 0.088 | 53 |
|  |  | C | 1.261 | 0.155 | 0.825 | 0.088 | 53 |
|  |  | AP | 0.099 | 0.013 | 0.082 | 0.005 | 20 |
|  |  | ML | 0.048 | 0.012 | 0.023 | 0.004 | 106 |
|  | Proportion >2 | VM | 14.239 | 5.685 | 1.049 | 1.091 | 1258 |
|  |  | C | 14.568 | 5.282 | 1.029 | 1.058 | 1316 |
|  |  | AP | 10.159 | 4.923 | 2.952 | 4.980 | 244 |
|  |  | ML | 4.846 | 3.464 | 0.646 | 0.506 | 651 |
|  | IQR | VM | 0.718 | 0.136 | 0.228 | 0.099 | 215 |
|  |  | C | 0.715 | 0.136 | 0.227 | 0.098 | 215 |
|  |  | AP | 0.064 | 0.012 | 0.032 | 0.010 | 98 |
|  |  | ML | 0.041 | 0.011 | 0.019 | 0.004 | 118 |
|  | SD | VM | 0.608 | 0.089 | 0.233 | 0.064 | 160 |
|  |  | C | 0.606 | 0.089 | 0.233 | 0.064 | 160 |
|  |  | AP | 0.061 | 0.009 | 0.028 | 0.005 | 120 |
|  |  | ML | 0.047 | 0.012 | 0.020 | 0.005 | 134 |
| L2/L3 | RMS | VM | 1.253 | 0.153 | 0.823 | 0.085 | 52 |
|  |  | C | 1.240 | 0.152 | 0.811 | 0.085 | 53 |
|  |  | AP | 0.171 | 0.020 | 0.139 | 0.007 | 23 |
|  |  | ML | 0.052 | 0.018 | 0.022 | 0.007 | 141 |
|  | Proportion >2 | VM | 14.661 | 5.181 | 1.119 | 1.097 | 1211 |
|  |  | C | 14.511 | 5.078 | 1.141 | 1.153 | 1172 |
|  |  | AP | 5.449 | 2.756 | 0.854 | 1.430 | 538 |
|  |  | ML | 7.938 | 6.197 | 0.744 | 0.521 | 967 |
|  | IQR | VM | 0.721 | 0.137 | 0.232 | 0.101 | 211 |
|  |  | C | 0.718 | 0.138 | 0.229 | 0.100 | 213 |
|  |  | AP | 0.081 | 0.015 | 0.039 | 0.013 | 106 |
|  |  | ML | 0.041 | 0.017 | 0.018 | 0.007 | 129 |
|  | SD | VM | 0.606 | 0.083 | 0.233 | 0.066 | 160 |
|  |  | C | 0.601 | 0.084 | 0.232 | 0.065 | 160 |
|  |  | AP | 0.083 | 0.013 | 0.035 | 0.008 | 138 |
|  |  | ML | 0.051 | 0.018 | 0.019 | 0.008 | 171 |
| L1/L2 | RMS | VM | 1.237 | 0.147 | 0.821 | 0.077 | 51 |
|  |  | C | 1.229 | 0.148 | 0.813 | 0.081 | 51 |
|  |  | AP | 0.129 | 0.019 | 0.106 | 0.030 | 21 |
|  |  | ML | 0.053 | 0.010 | 0.019 | 0.004 | 179 |
|  | Proportion >2 | VM | 13.749 | 4.999 | 1.020 | 1.021 | 1248 |
|  |  | C | 14.081 | 4.813 | 1.105 | 1.107 | 1174 |
|  |  | AP | 2.290 | 2.008 | 1.058 | 1.822 | 116 |
|  |  | ML | 13.841 | 6.509 | 1.829 | 1.332 | 657 |
|  | IQR | VM | 0.703 | 0.135 | 0.224 | 0.098 | 213 |
|  |  | C | 0.707 | 0.137 | 0.228 | 0.099 | 211 |
|  |  | AP | 0.082 | 0.043 | 0.041 | 0.020 | 101 |
|  |  | ML | 0.048 | 0.011 | 0.013 | 0.004 | 268 |
|  | SD | VM | 0.588 | 0.079 | 0.225 | 0.064 | 161 |
|  |  | C | 0.589 | 0.079 | 0.227 | 0.065 | 160 |
|  |  | AP | 0.089 | 0.020 | 0.041 | 0.015 | 119 |
|  |  | ML | 0.050 | 0.009 | 0.017 | 0.004 | 185 |
| T12/L1 | RMS | VM | 1.189 | 0.139 | 0.819 | 0.061 | 45 |
|  |  | C | 1.178 | 0.139 | 0.813 | 0.063 | 45 |
|  |  | AP | 0.134 | 0.020 | 0.088 | 0.032 | 53 |
|  |  | ML | 0.073 | 0.013 | 0.027 | 0.006 | 168 |
|  | Proportion >2 | VM | 10.183 | 4.003 | 0.609 | 0.525 | 1573 |
|  |  | C | 10.108 | 3.993 | 0.672 | 0.586 | 1404 |
|  |  | AP | 8.523 | 8.490 | 3.063 | 4.566 | 178 |
|  |  | ML | 15.188 | 7.059 | 1.798 | 1.464 | 745 |
|  | IQR | VM | 0.633 | 0.121 | 0.214 | 0.090 | 195 |
|  |  | C | 0.630 | 0.122 | 0.215 | 0.090 | 194 |
|  |  | AP | 0.127 | 0.073 | 0.055 | 0.024 | 132 |
|  |  | ML | 0.067 | 0.015 | 0.018 | 0.004 | 271 |
|  | SD | VM | 0.538 | 0.081 | 0.207 | 0.054 | 159 |
|  |  | C | 0.535 | 0.081 | 0.208 | 0.054 | 158 |
|  |  | AP | 0.123 | 0.024 | 0.054 | 0.023 | 129 |
|  |  | ML | 0.070 | 0.012 | 0.026 | 0.006 | 172 |
| T11/T12 | RMS | VM | 1.164 | 0.136 | 0.815 | 0.049 | 43 |
|  |  | C | 1.154 | 0.135 | 0.812 | 0.049 | 42 |
|  |  | AP | 0.129 | 0.027 | 0.063 | 0.027 | 105 |
|  |  | ML | 0.077 | 0.015 | 0.031 | 0.006 | 149 |
|  | Proportion >2 | VM | 7.509 | 3.188 | 0.472 | 0.366 | 1490 |
|  |  | C | 7.262 | 3.026 | 0.446 | 0.319 | 1527 |
|  |  | AP | 22.115 | 10.459 | 5.502 | 6.318 | 302 |
|  |  | ML | 14.506 | 7.859 | 1.708 | 1.525 | 749 |
|  | IQR | VM | 0.574 | 0.111 | 0.202 | 0.085 | 184 |
|  |  | C | 0.565 | 0.110 | 0.201 | 0.084 | 180 |
|  |  | AP | 0.141 | 0.072 | 0.055 | 0.024 | 155 |
|  |  | ML | 0.069 | 0.017 | 0.021 | 0.006 | 225 |
|  | SD | VM | 0.515 | 0.088 | 0.201 | 0.047 | 157 |
|  |  | C | 0.510 | 0.089 | 0.199 | 0.045 | 156 |
|  |  | AP | 0.124 | 0.022 | 0.053 | 0.024 | 132 |
|  |  | ML | 0.074 | 0.015 | 0.029 | 0.007 | 158 |
| T10/T11 | RMS | VM | 1.098 | 0.130 | 0.773 | 0.044 | 42 |
|  |  | C | 1.077 | 0.128 | 0.765 | 0.044 | 41 |
|  |  | AP | 0.202 | 0.039 | 0.100 | 0.035 | 101 |
|  |  | ML | 0.071 | 0.017 | 0.030 | 0.005 | 141 |
|  | Proportion >2 | VM | 6.541 | 3.088 | 0.454 | 0.345 | 1342 |
|  |  | C | 5.986 | 2.981 | 0.407 | 0.266 | 1369 |
|  |  | AP | 34.119 | 10.980 | 12.190 | 15.601 | 180 |
|  |  | ML | 12.736 | 7.148 | 1.218 | 0.914 | 945 |
|  | IQR | VM | 0.515 | 0.103 | 0.188 | 0.077 | 174 |
|  |  | C | 0.491 | 0.101 | 0.183 | 0.074 | 168 |
|  |  | AP | 0.187 | 0.066 | 0.062 | 0.029 | 203 |
|  |  | ML | 0.062 | 0.017 | 0.023 | 0.006 | 169 |
|  | SD | VM | 0.485 | 0.091 | 0.189 | 0.041 | 156 |
|  |  | C | 0.471 | 0.092 | 0.185 | 0.038 | 155 |
|  |  | AP | 0.146 | 0.018 | 0.059 | 0.026 | 147 |
|  |  | ML | 0.070 | 0.017 | 0.028 | 0.006 | 154 |
| T9/T10 | RMS | VM | 0.913 | 0.110 | 0.648 | 0.036 | 41 |
|  |  | C | 0.896 | 0.110 | 0.641 | 0.036 | 40 |
|  |  | AP | 0.169 | 0.028 | 0.091 | 0.025 | 85 |
|  |  | ML | 0.051 | 0.010 | 0.019 | 0.003 | 165 |
|  | Proportion >2 | VM | 5.814 | 2.957 | 0.435 | 0.217 | 1235 |
|  |  | C | 5.422 | 2.879 | 0.443 | 0.235 | 1125 |
|  |  | AP | 33.057 | 12.193 | 9.204 | 10.737 | 259 |
|  |  | ML | 15.092 | 5.459 | 2.067 | 1.837 | 630 |
|  | IQR | VM | 0.395 | 0.084 | 0.149 | 0.058 | 164 |
|  |  | C | 0.376 | 0.083 | 0.145 | 0.055 | 158 |
|  |  | AP | 0.146 | 0.044 | 0.052 | 0.022 | 181 |
|  |  | ML | 0.044 | 0.009 | 0.016 | 0.005 | 166 |
|  | SD | VM | 0.396 | 0.083 | 0.159 | 0.030 | 150 |
|  |  | C | 0.387 | 0.084 | 0.156 | 0.029 | 148 |
|  |  | AP | 0.113 | 0.011 | 0.047 | 0.019 | 139 |
|  |  | ML | 0.049 | 0.011 | 0.018 | 0.004 | 173 |
| T8/T9 | RMS | VM | 0.887 | 0.114 | 0.618 | 0.047 | 44 |
|  |  | C | 0.866 | 0.115 | 0.606 | 0.049 | 43 |
|  |  | AP | 0.188 | 0.028 | 0.116 | 0.023 | 62 |
|  |  | ML | 0.042 | 0.007 | 0.020 | 0.004 | 110 |
|  | Proportion >2 | VM | 5.697 | 2.944 | 0.474 | 0.256 | 1101 |
|  |  | C | 5.389 | 2.900 | 0.485 | 0.275 | 1010 |
|  |  | AP | 28.164 | 11.238 | 6.058 | 6.395 | 365 |
|  |  | ML | 13.792 | 7.031 | 4.181 | 3.682 | 230 |
|  | IQR | VM | 0.364 | 0.089 | 0.146 | 0.050 | 149 |
|  |  | C | 0.343 | 0.089 | 0.141 | 0.046 | 143 |
|  |  | AP | 0.145 | 0.040 | 0.053 | 0.023 | 175 |
|  |  | ML | 0.044 | 0.008 | 0.019 | 0.004 | 129 |
|  | SD | VM | 0.392 | 0.087 | 0.156 | 0.029 | 151 |
|  |  | C | 0.383 | 0.088 | 0.153 | 0.029 | 150 |
|  |  | AP | 0.113 | 0.011 | 0.049 | 0.018 | 131 |
|  |  | ML | 0.040 | 0.006 | 0.019 | 0.004 | 117 |
| T7/T8 | RMS | VM | 0.802 | 0.107 | 0.550 | 0.043 | 46 |
|  |  | C | 0.776 | 0.107 | 0.535 | 0.046 | 45 |
|  |  | AP | 0.196 | 0.027 | 0.125 | 0.020 | 56 |
|  |  | ML | 0.030 | 0.006 | 0.016 | 0.004 | 85 |
|  | Proportion >2 | VM | 5.682 | 3.075 | 0.478 | 0.231 | 1090 |
|  |  | C | 5.391 | 2.925 | 0.503 | 0.270 | 972 |
|  |  | AP | 24.785 | 12.077 | 3.548 | 4.772 | 599 |
|  |  | ML | 13.175 | 7.544 | 5.687 | 4.849 | 132 |
|  | IQR | VM | 0.321 | 0.084 | 0.131 | 0.043 | 145 |
|  |  | C | 0.297 | 0.086 | 0.126 | 0.040 | 136 |
|  |  | AP | 0.135 | 0.032 | 0.050 | 0.021 | 171 |
|  |  | ML | 0.031 | 0.006 | 0.016 | 0.007 | 90 |
|  | SD | VM | 0.359 | 0.083 | 0.142 | 0.026 | 153 |
|  |  | C | 0.350 | 0.084 | 0.139 | 0.026 | 151 |
|  |  | AP | 0.108 | 0.011 | 0.046 | 0.015 | 136 |
|  |  | ML | 0.029 | 0.006 | 0.015 | 0.004 | 92 |
| T6/T7 | RMS | VM | 0.697 | 0.089 | 0.475 | 0.037 | 47 |
|  |  | C | 0.666 | 0.089 | 0.454 | 0.037 | 47 |
|  |  | AP | 0.203 | 0.027 | 0.137 | 0.019 | 48 |
|  |  | ML | 0.036 | 0.006 | 0.017 | 0.002 | 109 |
|  | Proportion >2 | VM | 5.792 | 3.219 | 0.451 | 0.174 | 1184 |
|  |  | C | 5.466 | 3.180 | 0.465 | 0.165 | 1076 |
|  |  | AP | 19.975 | 11.846 | 2.300 | 3.733 | 769 |
|  |  | ML | 7.038 | 4.039 | 1.471 | 0.784 | 378 |
|  | IQR | VM | 0.268 | 0.065 | 0.109 | 0.031 | 145 |
|  |  | C | 0.247 | 0.066 | 0.103 | 0.025 | 139 |
|  |  | AP | 0.125 | 0.023 | 0.050 | 0.023 | 151 |
|  |  | ML | 0.027 | 0.005 | 0.016 | 0.006 | 70 |
|  | SD | VM | 0.313 | 0.070 | 0.128 | 0.020 | 145 |
|  |  | C | 0.302 | 0.071 | 0.124 | 0.020 | 143 |
|  |  | AP | 0.103 | 0.012 | 0.045 | 0.013 | 130 |
|  |  | ML | 0.034 | 0.006 | 0.015 | 0.003 | 124 |
| T5/T6 | RMS | VM | 0.714 | 0.092 | 0.482 | 0.046 | 48 |
|  |  | C | 0.677 | 0.092 | 0.456 | 0.048 | 49 |
|  |  | AP | 0.222 | 0.027 | 0.153 | 0.020 | 45 |
|  |  | ML | 0.030 | 0.007 | 0.015 | 0.005 | 99 |
|  | Proportion >2 | VM | 5.511 | 3.204 | 0.497 | 0.177 | 1009 |
|  |  | C | 5.212 | 3.141 | 0.507 | 0.158 | 929 |
|  |  | AP | 14.727 | 8.001 | 1.097 | 1.552 | 1243 |
|  |  | ML | 6.416 | 4.518 | 0.135 | 0.084 | 4636 |
|  | IQR | VM | 0.274 | 0.072 | 0.120 | 0.040 | 129 |
|  |  | C | 0.254 | 0.074 | 0.114 | 0.038 | 123 |
|  |  | AP | 0.122 | 0.019 | 0.050 | 0.025 | 144 |
|  |  | ML | 0.023 | 0.006 | 0.014 | 0.005 | 67 |
|  | SD | VM | 0.335 | 0.077 | 0.143 | 0.028 | 134 |
|  |  | C | 0.324 | 0.077 | 0.140 | 0.029 | 131 |
|  |  | AP | 0.104 | 0.013 | 0.045 | 0.012 | 132 |
|  |  | ML | 0.029 | 0.006 | 0.013 | 0.004 | 122 |
| T4/T5 | RMS | VM | 0.658 | 0.083 | 0.447 | 0.043 | 47 |
|  |  | C | 0.628 | 0.084 | 0.424 | 0.046 | 48 |
|  |  | AP | 0.193 | 0.022 | 0.140 | 0.020 | 37 |
|  |  | ML | 0.028 | 0.006 | 0.014 | 0.003 | 104 |
|  | Proportion >2 | VM | 5.199 | 3.276 | 0.486 | 0.143 | 969 |
|  |  | C | 5.137 | 3.238 | 0.515 | 0.137 | 898 |
|  |  | AP | 8.571 | 4.635 | 0.460 | 0.529 | 1762 |
|  |  | ML | 10.225 | 6.479 | 2.687 | 2.292 | 280 |
|  | IQR | VM | 0.244 | 0.067 | 0.115 | 0.037 | 112 |
|  |  | C | 0.230 | 0.069 | 0.111 | 0.037 | 106 |
|  |  | AP | 0.091 | 0.013 | 0.049 | 0.023 | 87 |
|  |  | ML | 0.025 | 0.005 | 0.013 | 0.005 | 83 |
|  | SD | VM | 0.312 | 0.074 | 0.138 | 0.027 | 126 |
|  |  | C | 0.304 | 0.075 | 0.136 | 0.029 | 124 |
|  |  | AP | 0.084 | 0.009 | 0.040 | 0.010 | 109 |
|  |  | ML | 0.027 | 0.005 | 0.013 | 0.004 | 108 |
| T3/T4 | RMS | VM | 0.622 | 0.068 | 0.428 | 0.034 | 45 |
|  |  | C | 0.585 | 0.070 | 0.399 | 0.036 | 47 |
|  |  | AP | 0.204 | 0.025 | 0.152 | 0.026 | 35 |
|  |  | ML | 0.036 | 0.008 | 0.017 | 0.004 | 107 |
|  | Proportion >2 | VM | 4.946 | 3.462 | 0.467 | 0.118 | 960 |
|  |  | C | 4.766 | 3.332 | 0.480 | 0.114 | 893 |
|  |  | AP | 6.714 | 3.904 | 0.362 | 0.265 | 1756 |
|  |  | ML | 12.913 | 7.116 | 3.853 | 1.951 | 235 |
|  | IQR | VM | 0.215 | 0.055 | 0.107 | 0.033 | 102 |
|  |  | C | 0.200 | 0.056 | 0.101 | 0.033 | 98 |
|  |  | AP | 0.087 | 0.015 | 0.046 | 0.023 | 87 |
|  |  | ML | 0.032 | 0.007 | 0.016 | 0.003 | 91 |
|  | SD | VM | 0.282 | 0.069 | 0.127 | 0.024 | 121 |
|  |  | C | 0.272 | 0.070 | 0.123 | 0.026 | 121 |
|  |  | AP | 0.085 | 0.010 | 0.041 | 0.011 | 105 |
|  |  | ML | 0.034 | 0.008 | 0.016 | 0.005 | 114 |
| T2/T3 | RMS | VM | 0.565 | 0.068 | 0.394 | 0.035 | 43 |
|  |  | C | 0.522 | 0.074 | 0.364 | 0.046 | 44 |
|  |  | AP | 0.209 | 0.025 | 0.145 | 0.029 | 44 |
|  |  | ML | 0.034 | 0.007 | 0.014 | 0.003 | 136 |
|  | Proportion >2 | VM | 4.596 | 3.509 | 0.498 | 0.145 | 822 |
|  |  | C | 4.549 | 3.607 | 0.535 | 0.164 | 751 |
|  |  | AP | 5.584 | 3.403 | 0.368 | 0.149 | 1418 |
|  |  | ML | 12.674 | 6.895 | 2.732 | 1.916 | 364 |
|  | IQR | VM | 0.179 | 0.043 | 0.092 | 0.022 | 96 |
|  |  | C | 0.165 | 0.044 | 0.084 | 0.023 | 96 |
|  |  | AP | 0.082 | 0.015 | 0.042 | 0.020 | 94 |
|  |  | ML | 0.025 | 0.005 | 0.011 | 0.003 | 121 |
|  | SD | VM | 0.254 | 0.075 | 0.117 | 0.023 | 117 |
|  |  | C | 0.243 | 0.075 | 0.113 | 0.024 | 116 |
|  |  | AP | 0.087 | 0.013 | 0.041 | 0.008 | 110 |
|  |  | ML | 0.033 | 0.007 | 0.013 | 0.003 | 162 |
| T1/T2 | RMS | VM | 1.189 | 0.139 | 0.819 | 0.061 | 45 |
|  |  | C | 0.399 | 0.096 | 0.290 | 0.068 | 38 |
|  |  | AP | 0.213 | 0.023 | 0.143 | 0.026 | 49 |
|  |  | ML | 0.045 | 0.017 | 0.019 | 0.004 | 138 |
|  | Proportion >2 | VM | 10.183 | 4.003 | 0.609 | 0.525 | 1573 |
|  |  | C | 4.794 | 3.682 | 0.739 | 0.424 | 548 |
|  |  | AP | 5.884 | 3.363 | 0.593 | 0.267 | 892 |
|  |  | ML | 9.188 | 6.806 | 3.166 | 2.725 | 190 |
|  | IQR | VM | 0.633 | 0.121 | 0.214 | 0.090 | 195 |
|  |  | C | 0.125 | 0.038 | 0.068 | 0.021 | 84 |
|  |  | AP | 0.078 | 0.014 | 0.045 | 0.018 | 74 |
|  |  | ML | 0.029 | 0.006 | 0.017 | 0.003 | 68 |
|  | SD | VM | 0.538 | 0.081 | 0.207 | 0.054 | 159 |
|  |  | C | 0.208 | 0.082 | 0.099 | 0.021 | 110 |
|  |  | AP | 0.095 | 0.021 | 0.048 | 0.012 | 96 |
|  |  | ML | 0.044 | 0.016 | 0.018 | 0.003 | 145 |

RMS = root mean square; >2 = proportion over twice walking; IQR = interquartile range; SD = standard deviation; VM = vector magnitude; C = compression; AP = anteroposterior shear; ML = mediolateral shear.

**Table G. General linear model coefficient estimates for net lumbosacral joint reaction forces.**

| Axis | Response | Predictor | Coefficient Estimate | Standard Error | t-statistic | P-value |
| --- | --- | --- | --- | --- | --- | --- |
| VM | log(RMS) | (Intercept) | 0.607 | 0.257 | 2.362 | 0.042 |
|  |  | Age | 0.002 | 0.004 | 0.581 | 0.576 |
|  |  | Body Mass | -0.002 | 0.004 | -0.453 | 0.662 |
|  |  | Sex | 0.072 | 0.072 | 0.997 | 0.345 |
|  |  | Group | -0.382 | 0.084 | -4.527 | 0.001 |
|  | log(Proportion >2) | (Intercept) | 2.831 | 1.248 | 2.268 | 0.050 |
|  |  | Age | 0.022 | 0.018 | 1.207 | 0.258 |
|  |  | Body Mass | -0.018 | 0.017 | -1.032 | 0.329 |
|  |  | Sex | -0.184 | 0.352 | -0.525 | 0.613 |
|  |  | Group | -2.697 | 0.410 | -6.581 | 0.000 |
|  | log(IQR) | (Intercept) | -0.151 | 0.571 | -0.264 | 0.798 |
|  |  | Age | 0.014 | 0.008 | 1.754 | 0.113 |
|  |  | Body Mass | -0.007 | 0.008 | -0.825 | 0.431 |
|  |  | Sex | 0.051 | 0.161 | 0.316 | 0.759 |
|  |  | Group | -1.058 | 0.187 | -5.646 | 0.000 |
|  | log(SD) | (Intercept) | -0.184 | 0.384 | -0.478 | 0.644 |
|  |  | Age | 0.009 | 0.005 | 1.628 | 0.138 |
|  |  | Body Mass | -0.005 | 0.005 | -1.019 | 0.335 |
|  |  | Sex | 0.115 | 0.108 | 1.061 | 0.317 |
|  |  | Group | -0.844 | 0.126 | -6.692 | 0.000 |
| C | log(RMS) | (Intercept) | 0.574 | 0.256 | 2.238 | 0.052 |
|  |  | Age | 0.002 | 0.004 | 0.585 | 0.573 |
|  |  | Body Mass | -0.002 | 0.004 | -0.436 | 0.673 |
|  |  | Sex | 0.061 | 0.072 | 0.842 | 0.422 |
|  |  | Group | -0.380 | 0.084 | -4.513 | 0.001 |
|  | log(Proportion >2) | (Intercept) | 2.882 | 1.192 | 2.418 | 0.039 |
|  |  | Age | 0.021 | 0.017 | 1.205 | 0.259 |
|  |  | Body Mass | -0.018 | 0.017 | -1.095 | 0.302 |
|  |  | Sex | -0.185 | 0.336 | -0.553 | 0.594 |
|  |  | Group | -2.738 | 0.391 | -7.000 | 0.000 |
|  | log(IQR) | (Intercept) | -0.200 | 0.569 | -0.351 | 0.734 |
|  |  | Age | 0.015 | 0.008 | 1.793 | 0.107 |
|  |  | Body Mass | -0.006 | 0.008 | -0.804 | 0.442 |
|  |  | Sex | 0.036 | 0.160 | 0.225 | 0.827 |
|  |  | Group | -1.063 | 0.187 | -5.688 | 0.000 |
|  | log(SD) | (Intercept) | -0.221 | 0.386 | -0.574 | 0.580 |
|  |  | Age | 0.009 | 0.006 | 1.644 | 0.135 |
|  |  | Body Mass | -0.005 | 0.005 | -1.005 | 0.341 |
|  |  | Sex | 0.100 | 0.109 | 0.921 | 0.381 |
|  |  | Group | -0.849 | 0.127 | -6.704 | 0.000 |
| AP | log(RMS) | (Intercept) | -0.803 | 0.264 | -3.046 | 0.014 |
|  |  | Age | 0.002 | 0.004 | 0.559 | 0.590 |
|  |  | Body Mass | -0.002 | 0.004 | -0.637 | 0.540 |
|  |  | Sex | 0.238 | 0.074 | 3.212 | 0.011 |
|  |  | Group | -0.411 | 0.087 | -4.753 | 0.001 |
|  | log(Proportion >2) | (Intercept) | 3.493 | 1.243 | 2.809 | 0.020 |
|  |  | Age | 0.018 | 0.018 | 1.033 | 0.328 |
|  |  | Body Mass | -0.025 | 0.017 | -1.441 | 0.183 |
|  |  | Sex | -0.132 | 0.350 | -0.378 | 0.715 |
|  |  | Group | -2.469 | 0.408 | -6.051 | 0.000 |
|  | log(IQR) | (Intercept) | -1.397 | 0.577 | -2.422 | 0.039 |
|  |  | Age | 0.012 | 0.008 | 1.423 | 0.188 |
|  |  | Body Mass | -0.008 | 0.008 | -1.039 | 0.326 |
|  |  | Sex | 0.241 | 0.162 | 1.484 | 0.172 |
|  |  | Group | -1.007 | 0.189 | -5.318 | 0.000 |
|  | log(SD) | (Intercept) | -1.487 | 0.363 | -4.096 | 0.003 |
|  |  | Age | 0.008 | 0.005 | 1.447 | 0.182 |
|  |  | Body Mass | -0.006 | 0.005 | -1.273 | 0.235 |
|  |  | Sex | 0.296 | 0.102 | 2.897 | 0.018 |
|  |  | Group | -0.768 | 0.119 | -6.451 | 0.000 |
| ML | log(RMS) | (Intercept) | -2.173 | 0.441 | -4.924 | 0.001 |
|  |  | Age | -0.008 | 0.006 | -1.231 | 0.249 |
|  |  | Body Mass | -0.005 | 0.006 | -0.831 | 0.428 |
|  |  | Sex | 0.204 | 0.124 | 1.644 | 0.135 |
|  |  | Group | -0.647 | 0.145 | -4.464 | 0.002 |
|  | log(Proportion >2) | (Intercept) | 1.502 | 2.698 | 0.557 | 0.591 |
|  |  | Age | 0.023 | 0.039 | 0.595 | 0.566 |
|  |  | Body Mass | -0.012 | 0.038 | -0.309 | 0.764 |
|  |  | Sex | -0.068 | 0.760 | -0.089 | 0.931 |
|  |  | Group | -1.789 | 0.886 | -2.020 | 0.074 |
|  | log(IQR) | (Intercept) | -3.065 | 0.386 | -7.942 | 0.000 |
|  |  | Age | -0.006 | 0.006 | -1.130 | 0.288 |
|  |  | Body Mass | 0.006 | 0.005 | 1.065 | 0.315 |
|  |  | Sex | 0.170 | 0.109 | 1.567 | 0.152 |
|  |  | Group | -0.708 | 0.127 | -5.587 | 0.000 |
|  | log(SD) | (Intercept) | -2.484 | 0.511 | -4.857 | 0.001 |
|  |  | Age | -0.006 | 0.007 | -0.819 | 0.434 |
|  |  | Body Mass | -0.002 | 0.007 | -0.222 | 0.830 |
|  |  | Sex | 0.206 | 0.144 | 1.433 | 0.186 |
|  |  | Group | -0.732 | 0.168 | -4.362 | 0.002 |

RMS = root mean square; >2 = proportion over twice walking; IQR = interquartile range; SD = standard deviation; VM = vector magnitude; C = compression; AP = anteroposterior shear; ML = mediolateral shear.

**Table H. General linear model summary statistics for net lumbosacral joint reaction forces.**

| Axis | Response | Model | | Sum of Squares | DF | Mean Squares | F | P |
| --- | --- | --- | --- | --- | --- | --- | --- | --- |
| VM | log(RMS) | | Total | 0.778 | 13 | 0.06 |  |  |
|  |  | | Model | 0.629 | 4 | 0.157 | 9.522 | 0.003 |
|  |  | | Residual | 0.149 | 9 | 0.017 |  |  |
|  | log(Proportion >2) | | Total | 34.695 | 13 | 2.669 |  |  |
|  |  | | Model | 31.181 | 4 | 7.795 | 19.97 | 0 |
|  |  | | Residual | 3.513 | 9 | 0.39 |  |  |
|  | log(IQR) | | Total | 5.951 | 13 | 0.458 |  |  |
|  |  | | Model | 5.216 | 4 | 1.304 | 15.979 | 0 |
|  |  | | Residual | 0.735 | 9 | 0.082 |  |  |
|  | log(SD) | | Total | 3.657 | 13 | 0.281 |  |  |
|  |  | | Model | 3.325 | 4 | 0.831 | 22.475 | 0 |
|  |  | | Residual | 0.333 | 9 | 0.037 |  |  |
| C | log(RMS) | | Total | 0.76 | 13 | 0.058 |  |  |
|  |  | | Model | 0.611 | 4 | 0.153 | 9.29 | 0.003 |
|  |  | | Residual | 0.148 | 9 | 0.016 |  |  |
|  | log(Proportion >2) | | Total | 35.163 | 13 | 2.705 |  |  |
|  |  | | Model | 31.962 | 4 | 7.99 | 22.464 | 0 |
|  |  | | Residual | 3.201 | 9 | 0.356 |  |  |
|  | log(IQR) | | Total | 5.961 | 13 | 0.459 |  |  |
|  |  | | Model | 5.231 | 4 | 1.308 | 16.121 | 0 |
|  |  | | Residual | 0.73 | 9 | 0.081 |  |  |
|  | log(SD) | | Total | 3.668 | 13 | 0.282 |  |  |
|  |  | | Model | 3.332 | 4 | 0.833 | 22.358 | 0 |
|  |  | | Residual | 0.335 | 9 | 0.037 |  |  |
| AP | log(RMS) | | Total | 1.152 | 13 | 0.089 |  |  |
|  |  | | Model | 0.995 | 4 | 0.249 | 14.301 | 0.001 |
|  |  | | Residual | 0.157 | 9 | 0.017 |  |  |
|  | log(Proportion >2) | | Total | 31.919 | 13 | 2.455 |  |  |
|  |  | | Model | 28.434 | 4 | 7.109 | 18.36 | 0 |
|  |  | | Residual | 3.485 | 9 | 0.387 |  |  |
|  | log(IQR) | | Total | 5.986 | 13 | 0.46 |  |  |
|  |  | | Model | 5.236 | 4 | 1.309 | 15.713 | 0 |
|  |  | | Residual | 0.75 | 9 | 0.083 |  |  |
|  | log(SD) | | Total | 3.564 | 13 | 0.274 |  |  |
|  |  | | Model | 3.267 | 4 | 0.817 | 24.757 | 0 |
|  |  | | Residual | 0.297 | 9 | 0.033 |  |  |
| ML | log(RMS) | | Total | 2.388 | 13 | 0.184 |  |  |
|  |  | | Model | 1.949 | 4 | 0.487 | 9.988 | 0.002 |
|  |  | | Residual | 0.439 | 9 | 0.049 |  |  |
|  | log(Proportion >2) | | Total | 30.981 | 13 | 2.383 |  |  |
|  |  | | Model | 14.575 | 4 | 3.644 | 1.999 | 0.178 |
|  |  | | Residual | 16.407 | 9 | 1.823 |  |  |
|  | log(IQR) | | Total | 1.986 | 13 | 0.153 |  |  |
|  |  | | Model | 1.651 | 4 | 0.413 | 11.064 | 0.002 |
|  |  | | Residual | 0.336 | 9 | 0.037 |  |  |
|  | log(SD) | | Total | 2.763 | 13 | 0.213 |  |  |
|  |  | | Model | 2.173 | 4 | 0.543 | 8.292 | 0.004 |
|  |  | | Residual | 0.59 | 9 | 0.066 |  |  |

RMS = root mean square; >2 = proportion over twice walking; IQR = interquartile range; SD = standard deviation; VM = vector magnitude; C = compression; AP = anteroposterior shear; ML = mediolateral shear.
